# A Survey of Priority Livestock Diseases and Laboratory Diagnostic Needs of Animal Health Professionals and Farmers in Uganda

**DOI:** 10.3389/fvets.2021.721800

**Published:** 2021-09-23

**Authors:** Patrick Vudriko, Abel B. Ekiri, Isabella Endacott, Sitira Williams, Nyangi Gityamwi, Joseph Byaruhanga, Ruth Alafiatayo, Erik Mijten, Robert Tweyongyere, Gabriel Varga, Alasdair J. C. Cook

**Affiliations:** ^1^Research Center for Tropical Diseases and Vector Control Laboratory, Department of Veterinary Pharmacy, Clinical and Comparative Medicine, School of Veterinary Medicine and Animal Resources, College of Veterinary Medicine, Animal Resources and Biosecurity, Makerere University, Kampala, Uganda; ^2^Department of Veterinary Epidemiology and Public Health, School of Veterinary Medicine, University of Surrey, Guildford, United Kingdom; ^3^Zoetis-ALPHA Initiative, Zoetis, Zaventem, Belgium

**Keywords:** animal health, livestock, veterinary, laboratory, diagnostics, laboratory technologist, veterinary professionals, Uganda

## Abstract

**Background:** Despite the investments made in veterinary diagnostic laboratory service delivery in Uganda, the scope and level of utilization remains low. This study aimed to determine the priority livestock diseases for which farmers and animal health professionals require veterinary diagnostic laboratory services, document the perceptions and opinions of key stakeholders on veterinary diagnostic laboratory services, and determine the factors that influence the delivery and utilization of animal disease diagnostic services in Uganda.

**Methods:** A qualitative study approach involving a survey and key informant interviews was used to collect relevant data from four stakeholder groups: animal health workers, laboratory technologists and technicians, farmers, and key informants. The survey data were exported to excel, and descriptive statistics performed. The key informant interview recordings were transcribed, and thematic analysis performed.

**Results:** The most reported diseases and conditions for which diagnostic services were needed were hemoparasites (including East Coast fever, anaplasmosis, babesiosis, and trypanosomosis), viral (including Foot and mouth disease, lumpy skin disease, rift valley fever, and papillomatosis), bacteria (including brucellosis, colibacillosis, anthrax, leptospirosis, and paratuberculosis) and protozoa diseases (coccidiosis), endoparasites (helminths), and mastitis. The most common diagnostic laboratory tests requested by clients, but laboratories were unable to provide included: rapid tests for contagious bovine pleuropneumonia, Foot and mouth disease, Newcastle disease, acaricide analysis, culture and antimicrobial sensitivity test, serology, and complete blood count. The most frequently reported challenges to providing diagnostic laboratory services were poor or lack of relevant equipment, insufficient or lack of supplies and reagents, high cost of reagents, inadequate or lack of laboratory staff to perform tests, and inadequate training of laboratory staff.

**Conclusions:** This study highlighted the need to improve provision of laboratory diagnostic services to meet the prioritized diagnostic needs of farmers and animal health professionals. Increased intersectoral engagement and funding support from the private, industry, and government sectors is necessary to help address the observed challenges to provision of diagnostic laboratory services, including equipping of the laboratories, provision of supplies, and hiring and training of laboratory staff. Finally, the findings also suggest that the education of farmers and animal health workers on the value and benefits of laboratory diagnostic services may contribute to increase in sample submission and subsequent demand for diagnostic laboratory services.

## Introduction

Livestock are sources of income, food security and livelihood to over 70% of the households in Uganda. With a rising demand for livestock products in both domestic and regional markets, there is a growing need to support farmers to produce and get more income from the various livestock value chains. However, livestock disease outbreaks continue to affect the productivity of the livestock sector in Uganda. The most reported disease problems in ruminants include East Coast fever (ECF), anaplasmosis, babesiosis, trypanososmosis, Foot and mouth disease (FMD), contagious bovine pleuropneumonia (CBPP), brucellosis, helminth infections, and blackquarter (1–4). In poultry, the most reported diseases include Newcastle disease and coccidiosis (1, 5), while in swine, African swine fever is commonly reported (6, 7).

Although the government of Uganda and development partners have over the last 2 decades invested in supporting disease control and strengthening animal disease diagnostics through establishment of veterinary laboratories, most laboratories continue to struggle to provide diagnostic services while other laboratories have collapsed or are dormant. In Uganda, the importance of veterinary laboratories in disease control is well-enshrined in the Animal Disease Control Act (8). Over 6 regional veterinary laboratories and 2 national diagnostics laboratories have been supported through the Ministry of Agriculture, Animal Industry and Fisheries (MAAIF), and the Food and Agriculture Organization as part of efforts to strengthen surveillance, diagnosis and disease reporting (9). Despite the above investments, both the scope and level of utilization of veterinary diagnostic laboratory services in Uganda remains low. Challenges such as insufficient reagents, consumables, human resources to manage laboratories and diagnostic kits have been reported as bottle necks to sustainable laboratory diagnostic service delivery in Uganda (10).

Veterinary diagnostic laboratory service is one of the critical competences the World Organization for Animal Health (OIE) designates in evaluation of the quality of veterinary services at a country level (11). Diagnostic laboratories traditionally support animal health and production, thus guaranteeing return to investment by commercial farms through early disease detection and interventions (12). The Zoetis-ALPHA (African Livestock Productivity and Health Advancement) Initiative, aims to contribute to building sustainable veterinary diagnostic infrastructure in Uganda (13). Through this Initiative, three veterinary diagnostic laboratories have been supported in efforts to build sustainable veterinary diagnostic businesses that will ultimately drive the growth of the livestock sector in Uganda. A key observation from the Zoetis-ALPHA initiative activities which is in alignment with findings of Nakayima et al. (10) is that the currently available veterinary laboratories are struggling to provide and sustain quality services to clients due to various challenges. Overcoming of these challenges in the new supported laboratories and other existing veterinary diagnostic laboratories requires a thorough review and assessment of the current veterinary diagnostic infrastructure in Uganda. To address this gap, this study sought to improve our understanding of the most important livestock diseases and conditions for which diagnostic tools or tests are required. Subsequently, this would inform the key players in laboratory diagnostic service delivery of the most relevant diagnostic tests and kits to equip the new laboratories with. Secondly, this information would be used to determine changes and improvements to veterinary laboratories to increase client satisfaction, thus enhancing and sustaining laboratory businesses. Thirdly, the findings may aid in identifying strategic and crucial interventions to strengthen veterinary laboratory infrastructure in Uganda.

The specific objectives of this study were to: (i) determine the priority livestock diseases for which farmers and animal health professionals require veterinary diagnostic laboratory services, (ii) document the perceptions and opinions of key stakeholders on veterinary diagnostic laboratory services, (iii) determine the factors that influence the delivery and utilization of veterinary diagnostic laboratory services in Uganda, and (iv) identify ways how preexisting and emerging veterinary diagnostic laboratories can be strengthened to provide sustainable diagnostic services that meet the needs of the growing livestock value chains in Uganda.

## Materials and Methods

### Study Area

**Figure 1:**
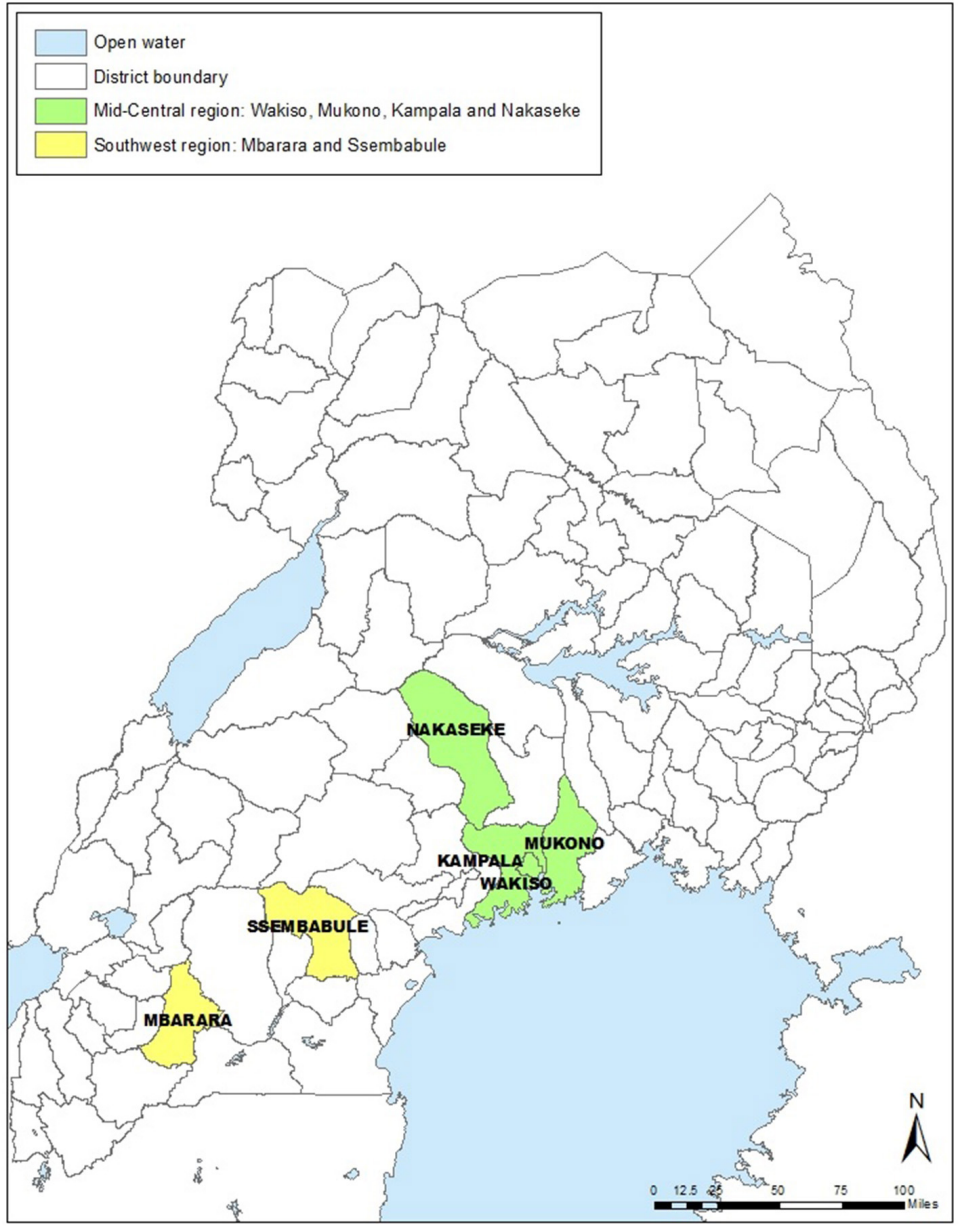
Map of Uganda showing study area. Mid-Central region: Wakiso, Mukono, Kampala, and Nakaseke districts. Southwest region: Mbarara and Ssembabule districts.

The mid-Central and Southwest regions of the country were purposively selected based on the predominant livestock species reared in these two regions and on the available budget to implement the study. The mid-Central region is among the regions with high poultry and piggery farming in Uganda. The districts surveyed in the mid-Central region included Wakiso, Mukono, Kampala and Nakaseke. The Southwest region, located farther from the capital city, Kampala, is among the regions with the highest number of commercial beef and dairy cattle farms and small ruminant production in the country. The districts surveyed in the Southwest region included Mbarara and Ssembabule; only two districts in this region were targeted due to limited funding (compared to four districts in the mid-central region). A total of 6 districts were targeted in this study (Figure 1); a table showing the number of livestock species by region and district (14) is provided in Supplementary Table 1 of Supplementary Data Sheet 1.

### Study Approach

A qualitative study approach was used to collect relevant data from four stakeholder groups: animal health workers, laboratory technologists and technicians, farmers, and key informants. The study sought to collect data on the opinions of these four participant groups; the use of a survey for animal health workers, laboratory technologists and technicians, and farmers, and key informant interviews for key informants were deemed appropriate in this study.

### Study Population

Four key stakeholder groups were targeted in this study and were selected based on their role in the veterinary diagnostic service value chain: animal health workers (veterinarians or veterinary officers, para-veterinarians, animal production officer, and assistant animal health officers), laboratory technologists and technicians, commercial farmers (commercial farmers involved in cattle, small ruminant, piggery and poultry production), and key informants. A total of 160 participants from the four stakeholder groups (animal health workers, laboratory technologists and technicians, farmers, and key informants) was estimated to participate in the study. The number of selected participants was determined based on available participants that met the inclusion criteria. Details of the number of participants from each stakeholder group and from each study region are presented in Supplementary Table 2 of Supplementary Data Sheet 1.

### Participant Enrollment and Selection Criteria

The lists of prospective study participants (animal health workers, laboratory technologists and technicians, farmers, and key informants) were compiled with the support of the District Veterinary Officers (DVOs). A list of DVOs in the six selected districts was obtained from the office of the Uganda Veterinary Association. The list of all active animal health workers obtained from the DVO's office was categorized according to type of practice, for example, ruminant, swine, small animal/companion, poultry, and mixed practice (multiple species). Key informants were purposively selected from each study district based on their experience and local knowledge of the veterinary clinical and diagnostic service industry.

#### Animal Health Workers

This participant group consisted of veterinary officers who were holders of a Bachelor of Veterinary Medicine degree, animal production officers who held a degree in animal production and management, and assistant animal health officers who were diploma holders in animal health and/or production. These three categories of veterinary human resource were purposively selected from across the study areas based on their level of involvement in clinical veterinary practice. Animal health workers who were not fully involved in clinical practice were excluded from the study.

#### Laboratory Technicians and Technologists

Only laboratory personnel that were duly qualified and were from recognized laboratory human resource training institutions were selected for the study. To be eligible the participant was either a laboratory technician with a certificate or held a diploma in laboratory science or was a laboratory technologist with a degree in laboratory sciences and worked in either a public or privately-owned veterinary laboratory.

#### Commercial Farmers

Consisted of commercial dairy, beef, poultry, and piggery farmers. The livestock enterprise selected varied between districts depending on the predominant livestock species that exist in each district. For instance, cattle are predominant in the Southwestern districts whereas poultry and piggery are the major livestock in Central Uganda. Emphasis was placed on selecting commercial farmers because demand for diagnostic services is expected to be highest for this category of farmers. We postulated that commercial farmers were more likely to have more extensive prior experiences (both negative and positive) regarding veterinary diagnostic services and as such capturing their responses would provide valuable information regarding the needs, challenges and diagnostic expectations of large-scale farmers.

#### Key Informants

The key informants included experienced managers of veterinary diagnostic laboratories, senior veterinary diagnosticians/clinicians, government officials working with MAAIF, farmers with experience in utilizing veterinary diagnostic services, and DVOs who provide oversight and supervision of veterinary laboratories.

### Data Collection Tools and Procedures

#### Animal Health Workers, Laboratory Technicians and Technologists, and Farmers

A questionnaire was designed and used to collect data from animal health workers, laboratory technologists and technicians, and farmers (see questionnaire tool in Supplementary Data Sheet 2). The type of data collected included demographic characteristics, perceptions of laboratory personnel on veterinary diagnostic services, laboratory diagnostic services offered by the veterinary laboratories, type of diagnostic tests requested by animal health workers and farmers, type of laboratory equipment and related challenges faced by laboratories, other challenges to provision of veterinary diagnostic services, submission of samples and factors that influence submission of samples to laboratories, solutions to the challenges affecting veterinary diagnostic laboratories, and level of satisfaction of animal health workers and farmers with veterinary diagnostic services. The survey was uploaded onto the electronic survey platform Qualtrics and administered using tablets by the local study team to the study participants.

The DVOs were contacted by one of the study investigators *via* telephone and notified twice of the planned visit of the study team to the district, at 2 and 1 weeks before the visit date. The potential study participants (animal health workers, laboratory technologists and technicians, and farmers) were contacted *via* telephone and notified of the date when the study team would meet them and conduct the survey. On the visit date, the purpose of the study was explained to each respondent and those who willing to participate were requested to provide informed consent electronically. To obtain the informed consent, each respondent was requested to read the purpose of the study on a tablet and asked to select his or her choice (agree or disagree). For respondents who did not read nor understand English, a study team member or local district veterinarian familiar with the local language helped in translation of the study background and the questionnaire. By clicking the agree button and giving consent, a respondent confirmed that: they had read and fully understood the purpose of the study; they voluntarily consent to participate in the study; and they belong to any of the following category of respondents: veterinarian, animal production officer, animal husbandry officer, laboratory technician/technologist or farmer. Upon consenting, the respondents were given the option to either proceed with answering the questionnaire on a tablet provided (self-administered) or get assistance from the research team to complete entry of responses on to the tablet. The completed surveys were saved and uploaded. Efforts were made to adhere to the standard operating procedures for COVID-19 prevention measures during survey administration; social distancing (standing or seating 2 meters apart) was observed, and masks and sanitizer were provided and used by the study team and study participants.

#### Key Informants

A key informant interview guide was designed and used to collect data from the selected key informants (see key informant guide in Supplementary Data Sheet 3). The interview questions were focused on common livestock diseases for which diagnostics are needed, challenges that affect the ability to use or offer diagnostic services, and solutions and recommendations as suggested by key informants. Potential key informants were contacted by telephone and questions were administered after consenting to the study.

### Data Analysis

The survey data was exported to excel, and descriptive statistics performed. Results were presented in tables and graphs. The key informant interview recording was transcribed and then analyzed. Thematic analysis method was used to analyze the data. An inductive approach was applied to examine patterns in the data (15), and the Braun and Clarke (16) 6 phase guideline was applied to analyze the data. The results of thematic analysis were categorized under each study question and codes were generated under each theme. The study questions focused on common livestock diseases for which diagnostics are needed, challenges that affect the ability to use or offer diagnostic services, and solutions and recommendations suggested by key informants. The resulting codes and themes were reported.

## Results

Survey results from the laboratory technologists/technicians, animal health workers and farmers, and results from the key informant interviews are presented in the sections below.

### Demographic Characteristics of the Study Participants

A total of 165 participants attempted the survey that was administered to laboratory technologists/technicians, animal health workers and farmers. Six of the 165 participants completed only the first question of the survey and were excluded from analysis, leaving a total of 159 respondents that were considered in the analyses. Farmers accounted for most of the respondents (86/159, 54%) followed by animal health workers (57/159, 36%) and laboratory technologists/technicians were the least represented (16/159, 10%) (Table 1). The total number of farmers, animal health workers, and laboratory personnel surveyed (*n* = 159) was different from that targeted (*n* = 148) as shown in Supplementary Table 2 of Supplementary Data Sheet 1 because an extra nine animal health workers and 4 laboratory personnel were identified by the DVOs and surveyed, and two farmers were not identified and therefore not surveyed.

**Table 1:** Demographic characteristics of respondents (farmers, laboratory technologists/technicians, and animal health workers).

**Variable**	**Response**	**All**	**Farmers**	**Lab personnel**	**AHWs**
		***n* = 159**	***n* = 86**	***n* = 16**	***n* = 57**
Gender	Male	115 (72.3)	56 (65.1)	12 (75.0)	47 (82.5)
	Female	44 (27.7)	30 (34.9)	4 (25.0)	10 (17.5)
Age (years)	≤25	6 (3.8)	4 (4.7)	1 (6.3)	1 (3.2)
	26–35	50 (31.4)	20 (23.3)	7 (43.8)	15 (48.4)
	36–45	49 (30.8)	27 (31.4)	2 (12.5)	11 (35.5)
	46–55	33 (20.7)	17 (19.8)	4 (25.0)	4 (12.9)
	≥56	21 (13.2)	17 (19.8)	2 (12.5)	0
	Prefer not to say	1 (0.6)	1 (1.2)	0	0
Workplace region	Ankole	25 (15.7)	12 (14)	3 (18.3)	10 (17.5)
	Busoga	1 (0.6)	0	0	0
	Kampala	26 (16.4)	8 (9.3)	9 (56.3)	9 (15.8)
	North Buganda	76 (47.8)	51 (59.3)	3 (18.8)	20 (35.1)
	South Buganda	31 (19.5)	13 (15.1)	1 (6.3)	15 (26.3)
	Teso	1 (0.6)	0	0	1 (1.8)
	Toro	1 (0.6)	0	0	1 (1.8)
	Others	3 (0.6)	2 (2.3)	0	1 (1.8)
Workplace districts	Amolatar	1 (0.6)	0	0	0
	Kabarole	1 (0.6)	0	0	1 (1.8)
	Kampala	18 (11.3)	5 (5.8)	7 (43.8)	6 (10.5)
	Luwero	1 (0.6)	0	0	1 (1.8)
	Mbarara	24 (15.1)	12 (13.9)	2 (12.5)	10 (17.5)
	Mukono	34 (21.4)	24 (27.9)	2 (12.5)	8 (14)
	Nakaseke	20 (12.6)	12 (13.9)	1 (6.3)	7 (12.3)
	Soroti	1 (0.6)	0	0	1 (1.8)
	Ssembabule	23 (14.5)	10 (11.6)	2 (12.5)	10 (17.5)
	Wakiso	38 (23.9)	23 (26.7)	2 (12.5)	13 (22.8)

*AHWs, Animal health workers; Data are presented as n (%)*.

Most of the respondents were male (115/159, 72%) and aged between 26 and 45 years old (99/159, 62%). The respondents worked mainly in the following districts: Wakiso (38/159, 24%), Mukono (34/159, 21%), Mbarara (24/159, 15%), Ssembabule (23/159, 14%), and Kampala (18/159, 11%) (Table 1).

The 16 laboratory personnel that participated in the study were employed in academia (Makerere University), government (national, regional and district laboratories) and privately-owned laboratories as shown in Supplementary Table 3 of Supplementary Data Sheet 1. Majority of the laboratory personnel had worked for between 0 and 10 years (11/16, 69%) although 3 of them worked for over 20 years.

Most of the animal health workers that participated in the study were employed by the government (39/57, 68%) while the rest worked in academic institutions and private practice. Ruminants, mixed practice and poultry were the most common type of veterinary practice for the participating animal health workers (Supplementary Table 4 of Supplementary Data Sheet 1).

Majority of the farmers were predominantly poultry (32/86, 37%), cattle (29/86, 34%), and pig (24/86, 28%) farmers, and more than half of all farmers reported keeping cattle and chicken at the time of survey. Over 70% (64/86) of the farmers had between 6 to over 20 years of experience in farming (Supplementary Table 5 of Supplementary Data Sheet 1).

A total of eight key informants were interviewed for the study. Details of the eight key informants interviewed are provided in Supplementary Data Sheet 4. Majority of the informants were veterinary surgeons (7/8, 87%) based in the following districts: Wakiso (1/8, 12%), Sembabule (1/8, 12%), Mbarara (1/8, 12%), Nakaseke (1/8, 12%), Kampala (Makerere: 1/8, 12% and MAAIF: 2/8, 25%) and one farmer (1/8, 12%) from Mukono district. To protect the informant's anonymity and confidentiality, their names were replaced with a code such as KII:01 which is an abbreviation for Key Informant Interview 1. Three themes were identified from the key informant data: clientele for veterinary diagnostic services, perspectives on challenges affecting veterinary laboratories, and solutions suggested by the veterinary sector. Relevant quotations were extracted from each of the eight key informants' transcripts. Details of the extracted quotations are provided in Supplementary Data Sheet 5.

### Perceptions of Laboratory Personnel on Veterinary Diagnostic Services

The laboratory personnel reported that both animal health workers (13/14, 93%) and farmers (12/14, 86%) appreciate the value of laboratory diagnostic services. When asked whether farmers are willing to pay for diagnostic services, 79% (11/14) of laboratory personnel reported that farmers are willing to pay. A similar proportion (79%) of respondents also believed veterinary diagnostic services can be run profitably (Supplementary Table 3 of Supplementary Data Sheet 1).

A half of the laboratory personnel reported that their laboratories had not carried out market surveys to understand the diagnostic needs of their clients. On a positive note, majority of the laboratory personnel (10/14, 71%) had attended continuous professional development (CPD) courses in the last 2 years on various topics shown in Supplementary Table 6 of Supplementary Data Sheet 1. Those that did not attend any CPD training cited lack of training opportunities within their organization.

### Laboratory Diagnostic Services Offered by the Veterinary Laboratories and Type of Diagnostic Tests Requested by Animal Health Workers and Farmers

To understand the capability of laboratories to provide diagnostic services, laboratory personnel were asked to indicate: the common diagnostic tests requested by clients, diagnostic tests requested but the laboratories were unable to provide, diagnostic tests they would like to have in their laboratories to improve the range of services offered to clients, commonly tested diseases, and the diseases or conditions laboratories would like to test but are currently unable to test.

**Figure 2:**
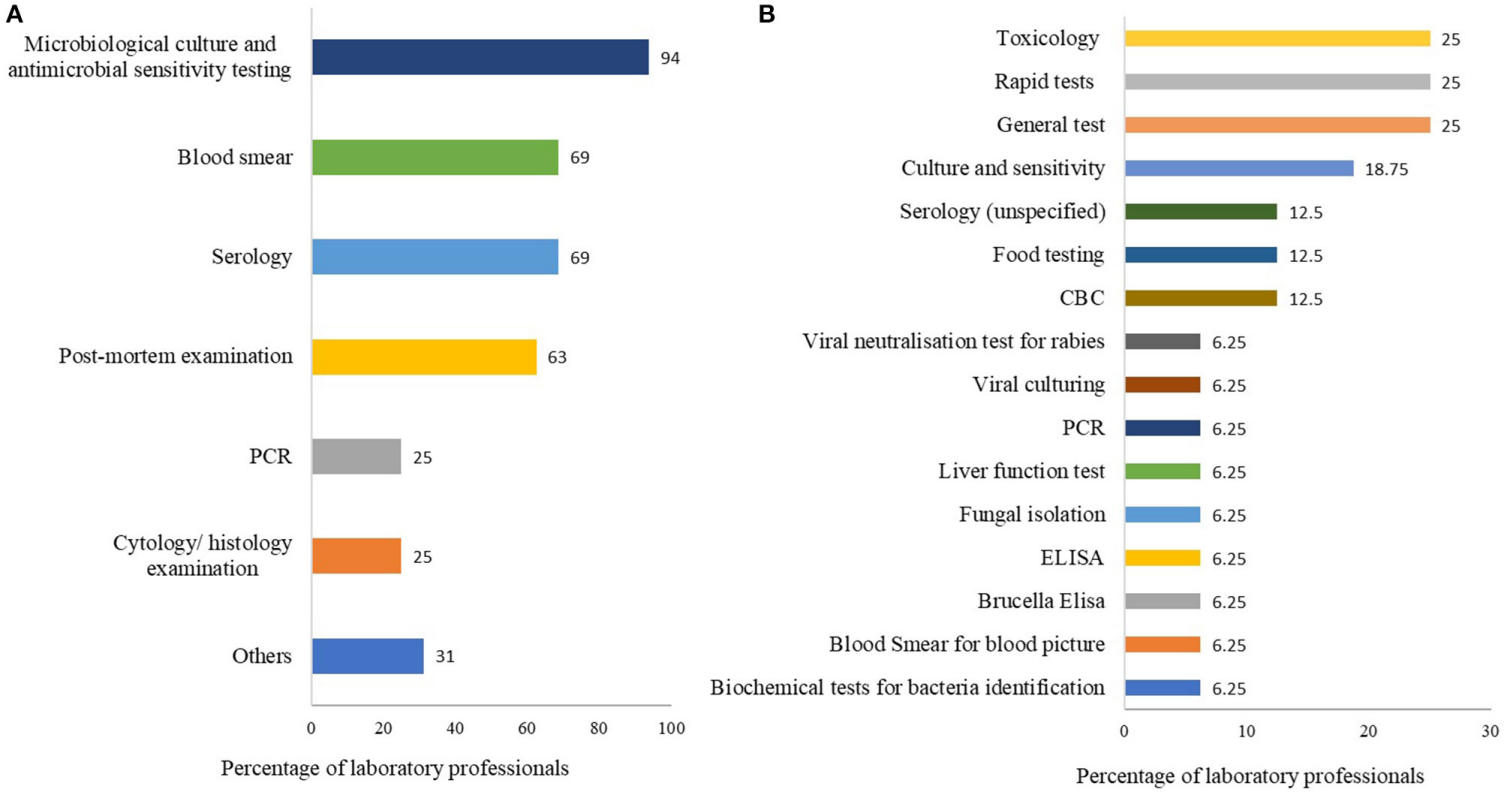
**(A,B)** The most common tests requested by clients. Graph **(A)** represents tests requested for which labs have capacity to test; **(B)** represents tests requested but laboratories have no capacity to tests. Both questions had multiple responses. Bars represent percent of laboratory technicians (*n* = 16) that selected each test.

The most common diagnostic tests requested were microbial culture and antimicrobial sensitivity tests (15/16, 94%), followed by serology (11/16, 69%), blood smear (11/16, 69%), post-mortem examination (10/16, 63%), complete blood count (9/16, 56%), and other tests as shown in Figure 2A. The top five diagnostic tests that were requested but laboratories were unable to provide included: rapid tests (for Contagious bovine pleuropneumonia (CBPP), Foot and mouth disease (FMD), Newcastle disease virus (NDV), and mycoplasma infection) (4/16, 25%); toxicological tests especially acaricide analysis (4/16, 25%); general gram staining (4/16, 25%); culture and antimicrobial sensitivity test (3/16, 19%) followed by serology and complete blood counting each at 13% (2/16) (Figure 2B).

**Figure 3:**
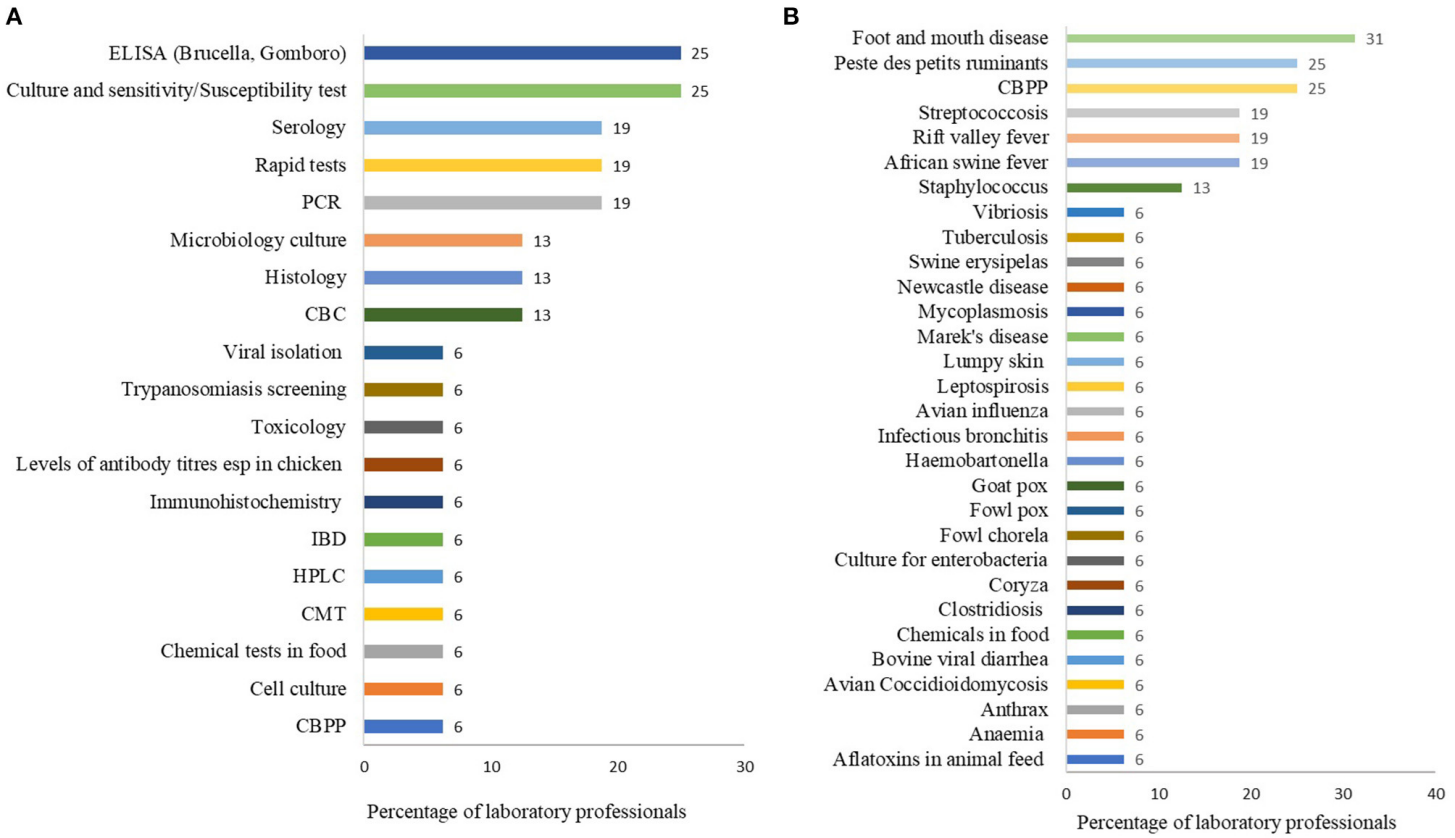
**(A)** Diagnostic tests that a laboratory would like to have in-order to improve the range of services offered to their clients. **(B)** Diseases or conditions that respondents would like to test at their laboratories but are currently unable to test. Both questions had multiple responses. Bars represent percent of laboratory technicians (*n* = 16) reporting a test or disease.

The diagnostic tests that laboratories would like to have to improve the range of services offered to clients were ELISA for Brucella and Gumboro (4/16, 25%), culture and sensitivity (4/16, 25%), serology for Black quarter, anthrax and viral diseases (4/16, 19%), rapid tests for viral diseases such as Newcastle disease (3/16, 19%) and PCR. The other tests mentioned are shown in Figure 3A.

**Figure 4:**
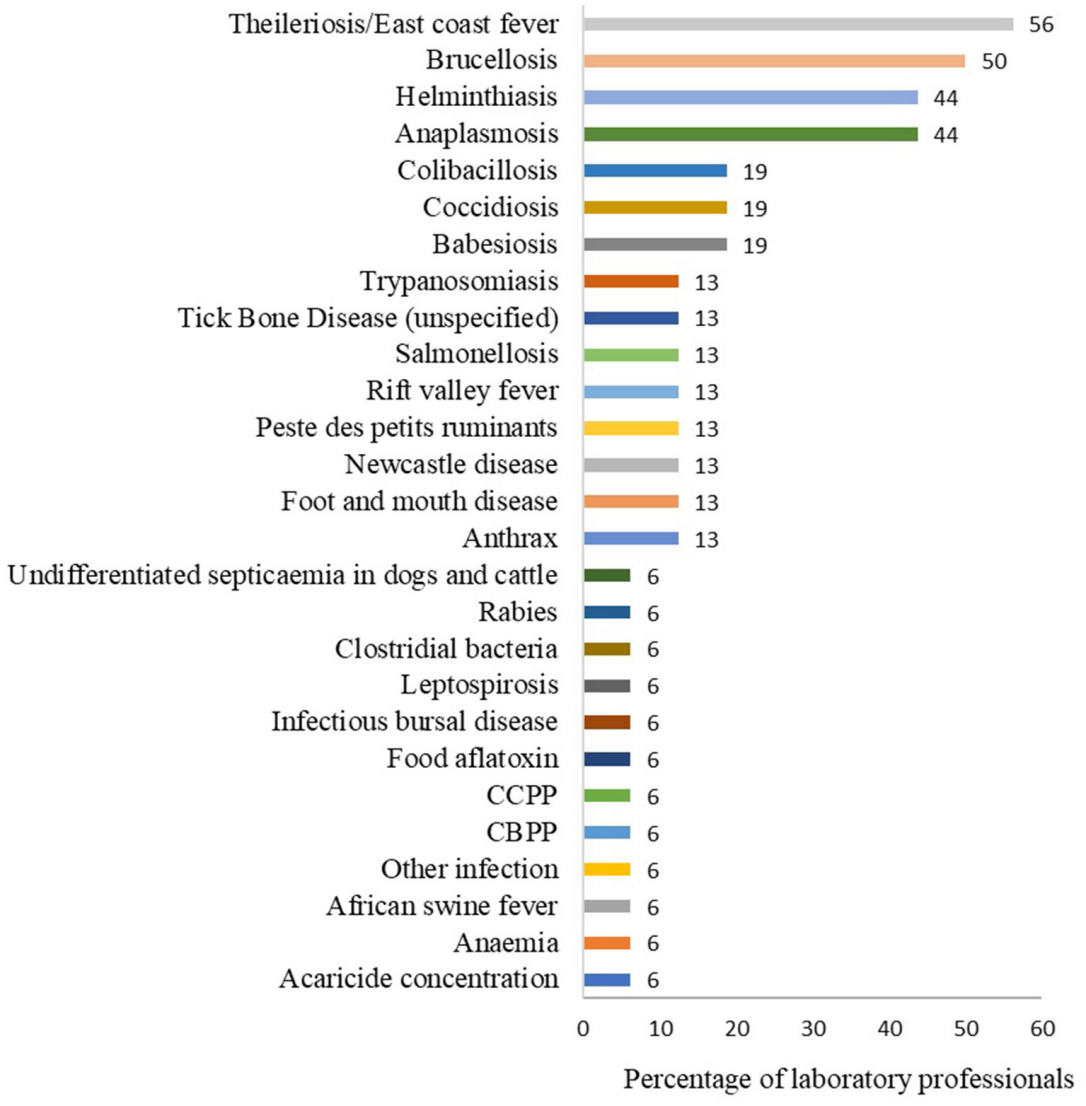
The most tested diseases in the laboratory. This question had multiple responses. Bars represent percent of laboratory technicians (*n* = 16) that selected each disease/condition.

The most frequently tested diseases were theileriosis (East coast fever) (9/16, 56%), brucellosis (8/16, 50%), helminthiasis and anaplasmosis each at 44% (9/16), colibacillosis, coccidiosis and babesiosis each at 19% (3/16) (Figure 4). Other diseases tested are shown in Figure 4. The top five diseases or conditions laboratories would like to test but are currently unable to test included FMD (5/16, 21%), PPR (4/16, 25%), CBPP (4/16, 25%), African swine fever (3/16, 19%), Rift valley fever (3/16, 19%), Streptococcus (3/16, 19%), and Staphylococcus infection (2/16, 13%) (Figure 3B). Other diseases are shown in Figure 3B.

**Figure 5:**
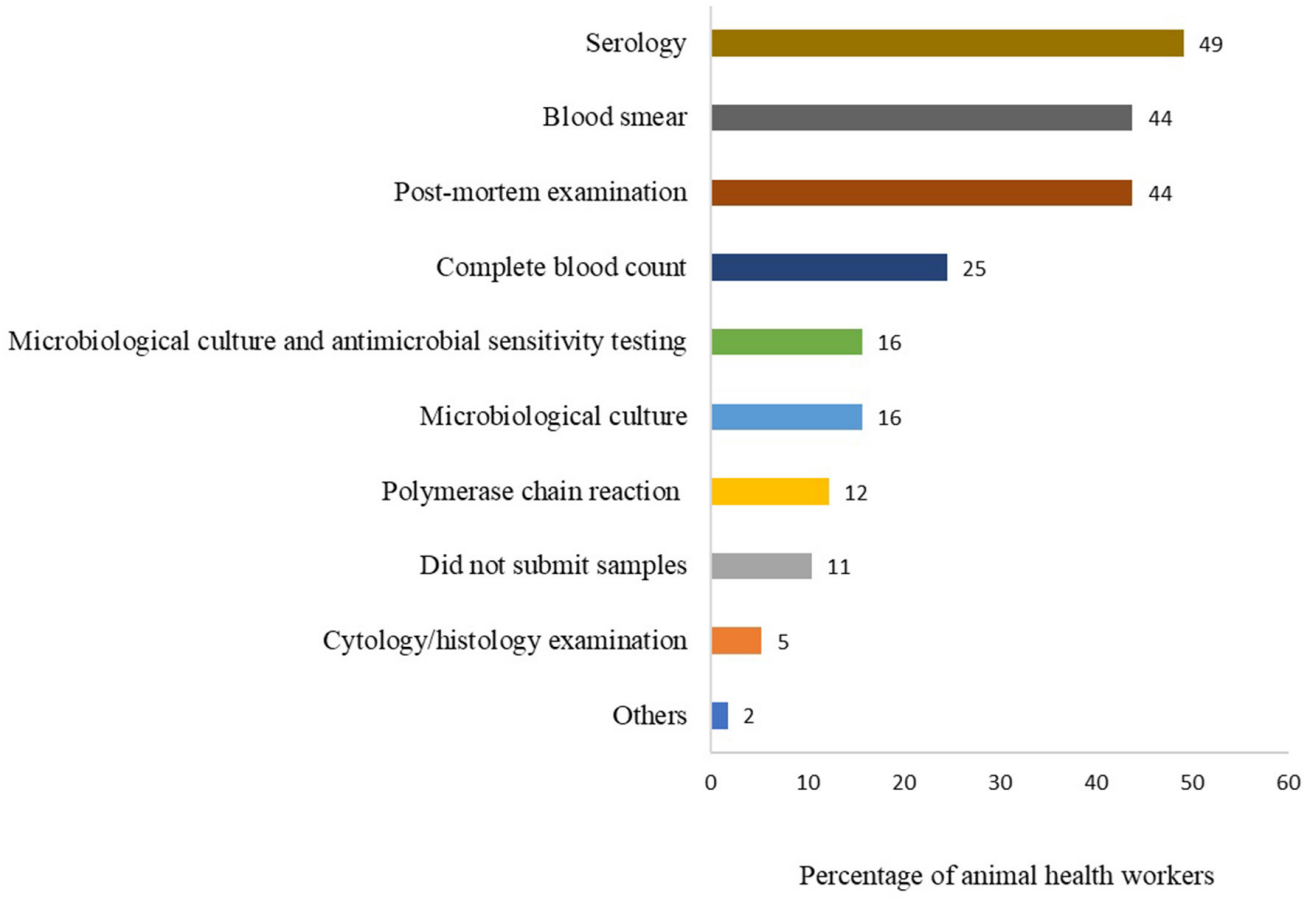
Type of tests commonly requested by animal health workers when submitting samples to laboratories for clinical diagnosis. This question had multiple responses. Bars represent percent of animal health workers (*n* = 57) that requested each type of test.

Animal health workers were asked the type of tests they commonly requested when submitting samples to the laboratory in the last 6 months. Serology test was the most frequently reported (28/57, 49%), followed by blood smear and post-mortem examination each reported by 44% (25/57) and other tests as shown in Figure 5.

One of the three themes identified from the key informant data was “clientele for veterinary diagnostic services.” The participating eight key informants were asked to “list the key clients which use laboratory animal diagnostic services” in Uganda and the most frequent responses were: “*farmers, commercial farmers, veterinarians, investors and researchers, co-operative societies, non-governmental organizations (NGOs), and community-based organizations (CBOs) like Mercy Corps, FAO, and pharmaceutical companies*.” The participants were then asked, “what are the diagnostic services which clients demand?” The direct quotations of various diseases and conditions listed by the eight key informants are shown in Box 1 below.

Box 1 **Common livestock/poultry diseases for which diagnostics are needed.**

**Table:** 

***KII:01**—“Lumpy skin disease, FMD, foot rot, blood parasites especially tick-borne parasites, viral diseases and mastitis.”*
***KII:03**—“Worm and TB (Para Tuberculosis), abortions and fetal deaths, FMD, Papilloma which has become a big problem, because we do not know whether we still have the real papilloma virus because there is some which is very aggressive, it can attack the whole herd.”*
***KII:03**—“Economic losses due to abortions and fertility challenges.”*
***KII:04**—“Brucellosis, sometimes FMD.”*
***KII:05**—“Tick borne diseases and pregnancy related diseases are very common.”*
***KII:06**—“CBPP, Brucellosis, African Swine Fever, Avian diseases.”*
***KII:07**—“Brucellosis, FMD, Anthrax, Rift Valley Fever, CBPP, Swine diseases, (ASF and all the other Hemorrhagic Syndromes, CSF, BSE and porcine erysipelas).”*
***KII:08**—“ECF, Trypanosomosis, Leptospirosos, Mastitis, fertility related diseases.”*

*KII, Key informant interview*.

### Laboratory Equipment and Related Challenges Faced by Laboratories

To assess the available functioning equipment, the laboratory personnel were asked to list the instruments or equipment most often used in their laboratories and the calibration status of the equipment where appropriate. The most frequently used equipment included microscope (12/16, 75%), fridges/freezer (8/12, 50%), pipettes, incubator, centrifuge each at 44% (7/16), autoclave (5/16, 31%), biosafety cabinet and weighing balance each at 25% (4/16). Other equipment included ELISA reader for serology (2/16), PCR machine (2/16) for gene amplification, HPLC for chemical analysis (1/16), auto-tissue processor (1/16), and microtome (1/16) for histopathological slide processing (Supplementary Table 7 of Supplementary Data Sheet 1).

Regarding calibration status of the equipment, majority (9/14) of the respondents reported that some equipment had been calibrated in the last 2 years. However, 36% (5/14) of laboratory personnel noted that equipment in their laboratories were not calibrated. Eight laboratory personnel further noted that service of the equipment in their laboratories was not performed, and two of the eight respondents reported that equipment service was not considered a priority in their laboratories. Other reported reasons for lack of equipment service were lack of finances (2/8), lack of equipment service policy (1/8) and servicing of equipment was postponed because of COVID-19 (1/8). Two respondents noted that the equipment was not due for service.

### Other Challenges to Provision of Veterinary Diagnostic Services

The laboratory technicians were asked to list and briefly explain the challenges to providing diagnostic services by their laboratories. The top five challenges reported by 81.3% (13/16) of the respondents included: lack of laboratory equipment, lack of human capacity to perform tests, poor sample quality, high cost of diagnostic services coupled with insufficient funding to run the laboratories, lack of or insufficient laboratory supplies, and lack of political support for veterinary laboratory diagnostic development (Table 2).

**Table 2:** The challenges that affect the ability to use or offer diagnostic services as reported by laboratory personnel (*n* = 13).

**Themes of challenges affecting veterinary diagnostic laboratories**	**Frequency**
Lack of laboratory equipment	9
Lack/inadequate technical skills to perform diagnostic tasks efficiently	5
Poor sample quality, sample collection, and storage techniques	4
High cost of diagnostic services, lack of or insufficient funding to run the laboratory services	4
Lack of, inadequate and untimely supply of test reagents and other diagnostic supplies	4
Lack of political support for laboratory diagnostic development	4
Poor management/administration of the laboratory	3
Under-utilization of laboratory services if available (low sample submission)	3
Poor access to laboratories (hard to reach laboratory, poor transport means)	3
Lack of equipment maintenance and calibration policy	3
Awareness of laboratory services by farmer is low (need for sensitization)	2
Unreliable power supply	2
Poor laboratory biosafety	2
Challenges in sourcing laboratory supplies	1

One of the three themes identified from the key informant data was “perspectives on challenges affecting veterinary laboratories.” The response of the key informants on challenges affecting veterinary diagnostic laboratories generated 4 codes (Codes 1–4). The codes within this theme provide an overview of factors which impact client access and the quality of diagnostic services provided. Code 1: access to laboratories, included “inadequate or lack of laboratory facilities,” “long distances to laboratories” and “poor access to laboratories.” Code 2: self-treatment on farms, included “treatment without diagnosis,” “animal losses,” and “preventative treatment.” Code 3: laboratory human resources, included “inadequate or lack of laboratory staff” and “inadequately trained staff.” The most common code in this theme was “access to laboratories,” participants referenced more about “access to laboratories” than the other two codes. Code 4: related to challenges of laboratory equipment and supplies and this included “poor equipping of laboratories,” “lack of relevant equipment,” “lack of reagents,” and “high cost of reagents,” all of which affect veterinary diagnostic service delivery. The direct quotations from the key informants are shown in Box 2.

Box 2 **Quotes from key informants on the challenges to delivery of veterinary diagnostic services.**

**Table:** 

**Code 1. Access to laboratories**	**Code 2. Self-treatment on farms**	**Code 3. laboratory human resources**	**Code 4. Laboratory equipment and supplies**
***KII:01**—*“*Veterinarians have to collect a sample, maybe cover another 30 miles to the location makes it more difficult*.” ***KII:02**—*“*We have only one laboratory in Mukono but it needs a farmer to travel quite a long distance to access services*.” ***KII:03**—*“*Accessibility to farmers is important, farmers call for help, and someone never comes so they get disappointed and sell off the animals*.” ***KII:04**—“Services are not run where there are very much required, i.e., in the villages where the farmers are located*.”	***Kll:01**—*“*Even if you say let's carry out the diagnosis and confirm, the veterinarian still wants to treat, instead of saying let me delay treatment for like an hour*.” ***Kll:02**—*“*8/10 of service providers will never advise a farmer on laboratory use before treatment. They use signs and symptoms to treat*.” ***Kll:03**—*“*when a farmer abuses self-treatment practices he faces losses, the animals die and next time he will call for a professional after feeling the shock of seeing his animals die*.” ***Kll:05**—*“*Farmers attitude they usually go with preventative treatment. They do not wait for animals to get sick*.” ***Kll:06**—*“*Users have spent a long time without using laboratories, so gravitating to using their own perceptions in managing animal diseases and products is the new norm*.”	***KII:01**—*“*The staff structure of the laboratory is still lacking, laboratories have laboratory technicians instead of laboratory technologists, who are more competent. In most cases you find that the CAO (Chief administrative officer) is not a scientist, so funding becomes a challenge*.” ***KII:02**—*“*Inadequate staff here in Mukono where we have one staff if he is on leave, sick or joins another organization, the service won't be sustained*.” ***KII:03**—*“*In the Nabitanga laboratory there is only one technician, you will find him overwhelmed when he goes for a workshop, no one is available to attend to the farmers*.” ***KII:06**—*“*We also don't have the right human resources even in numbers, in the district laboratories which don't have personnel. The veterinarian or DVO (district veterinary officer) is the laboratory technician. Even government lab there is lack of staff*.”	***KII:01**—*“*Laboratory supplies, equipment, reagents is arounds below 30%, the district labs are poorly equipped compared to the national or regional laboratories*.” ***KII:03**—*“*We are lacking equipment and technology, the problem is consistency on and off challenges, you find this time they can do Leptospirosis and next time they do not have reagents*.” ***KII:04**—*“*We lack reagents here, there are some kits or reagents which are very expensive and you find it very difficult because we can only examine a few animals with the kits that we have*.” ***KII:06**—*“*Our national level of equipment is lacking because at some point we constructed BSL3 laboratory (NADEC) but has never operated one*.” ***KII:07**—*“*We supply them with kits for serology but still as I told you the services are free so we do not meet their needs 100%*.”

*KII, Key informant interview*.

### Submission of Samples and Factors That Influence Submission of Samples to Laboratories

Given the experience of the laboratory personnel in veterinary diagnostic services, they were asked their opinion on the factors that influence clients to submit samples to a laboratory and to rank the factors by importance. The most frequently reported factors included: sample turn-around time (13/14, 93%), professionalism (skillfulness exhibited) (13/14, 93%), respect and appreciation of laboratory staff toward clients (13/14, 93%), laboratory staff availability (12/14, 86%), available diagnostic tests (12/14, 86%), guidance and communication (12/14, 86%), and other factors.

**Figure 6:**
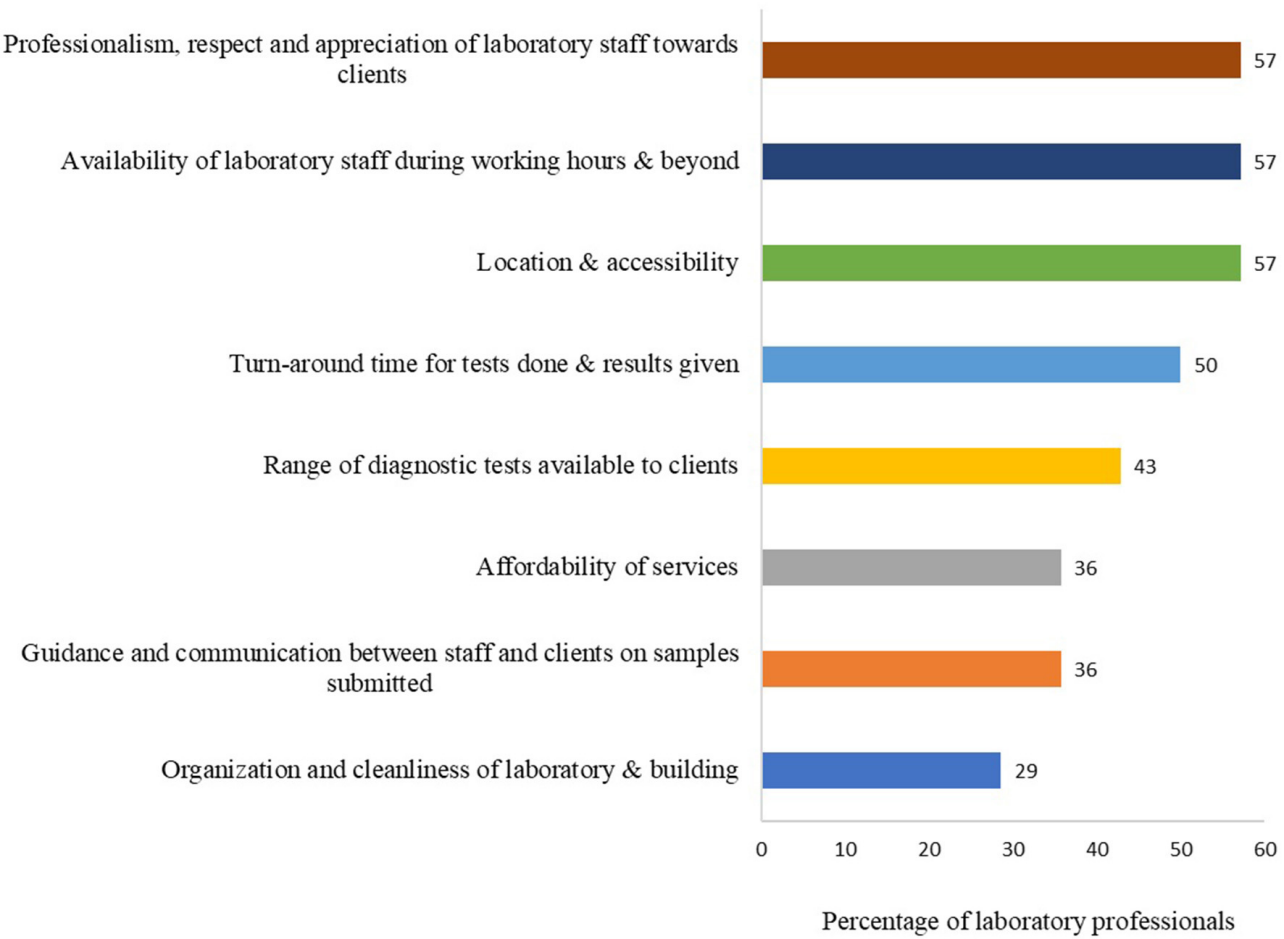
The top five important factors that determine whether clients will submit samples to the laboratory. Bars represent percent of laboratory technicians (*n* = 14) that selected each factor. This question had multiple responses.

The top five ranked factors based on importance included: professionalism, respect and appreciation of laboratory staff toward clients (8/14, 57%), availability of laboratory staff during working hours and beyond (8/14, 57%), location and accessibility (proximity of laboratory to their farm premises (8/14, 57%), turn-around time for tests performed and results given (7/14, 50%), and range of diagnostic tests available to clients (6/14, 43%) (Figure 6).

**Figure 7:**
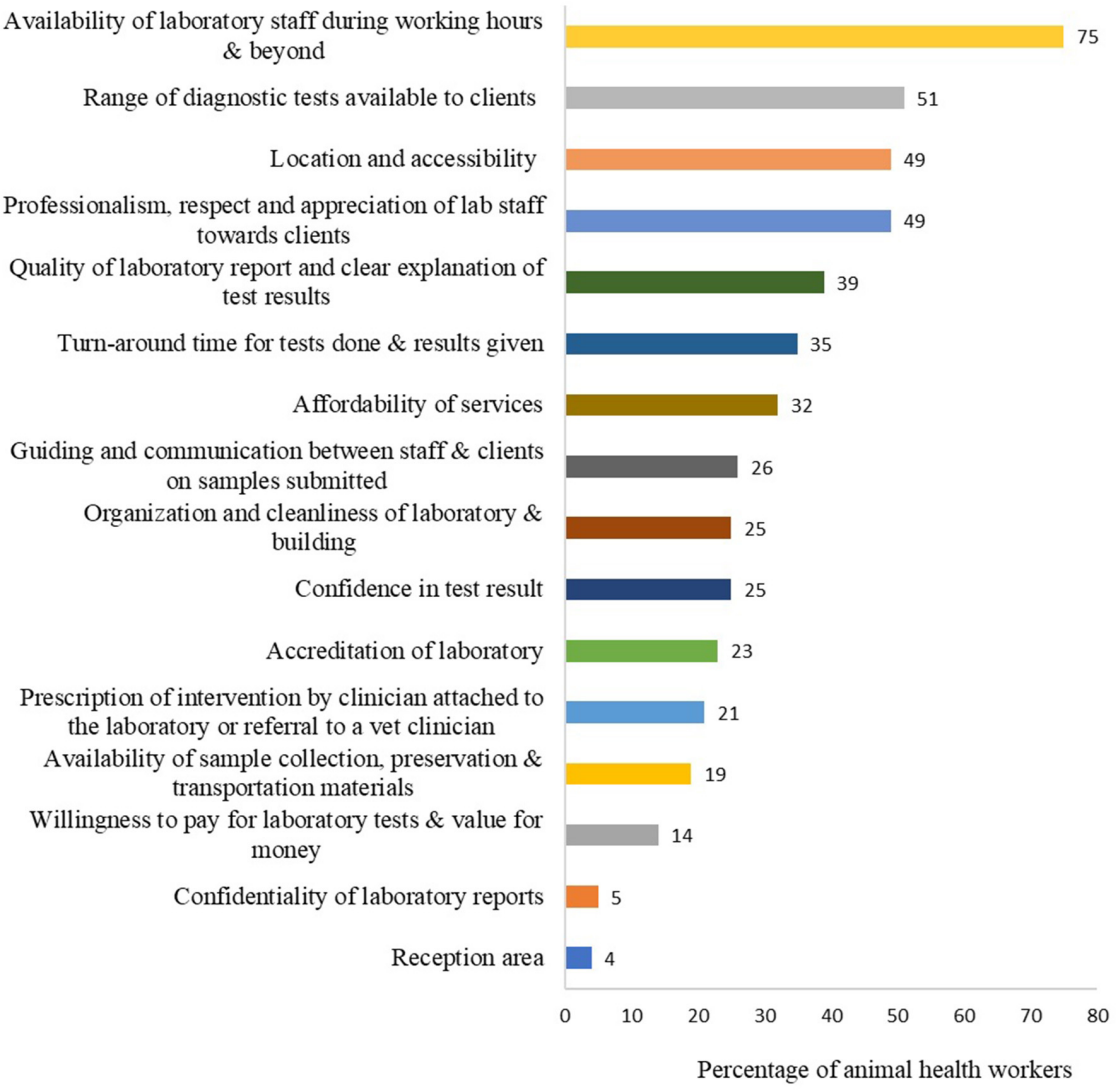
Top ranked important factors that determine whether animal health workers would submit samples to a laboratory or not. Bars represent percent of animal health workers (*n* = 57) that selected each factor. This question had multiple responses.

About 86% (49/57) of the animal health workers reported submitting samples to the laboratory at least once in the 6 months prior to the survey. Only 21% (12/57) of the respondents submitted samples over three times a month to the laboratory. Samples from cattle (42/57, 74%), goats (23/57, 40%) and chicken (17/57, 30%) were the most frequently submitted by animal health workers (Supplementary Table 4 of Supplementary Data Sheet 1). Animal health workers were asked the factors that influence their decision to submit samples to a laboratory and to rank the factors in order of importance. The top factors included availability of laboratory staff during working hours and beyond (43/57, 75%), range of diagnostic tests available to clients (29/57, 51%), location and accessibility (proximity of laboratory to farm premises) (28/57, 49%), professionalism, respect and appreciation of laboratory staff toward clients (28/57, 49%), quality of laboratory report (completeness and comprehension, 22/57, 39%), and sample turn-around time (20/57, 35%) (Figure 7). Other factors are presented in Figure 7 and Supplementary Table 8 of Supplementary Data Sheet 1.

**Figure 8:**
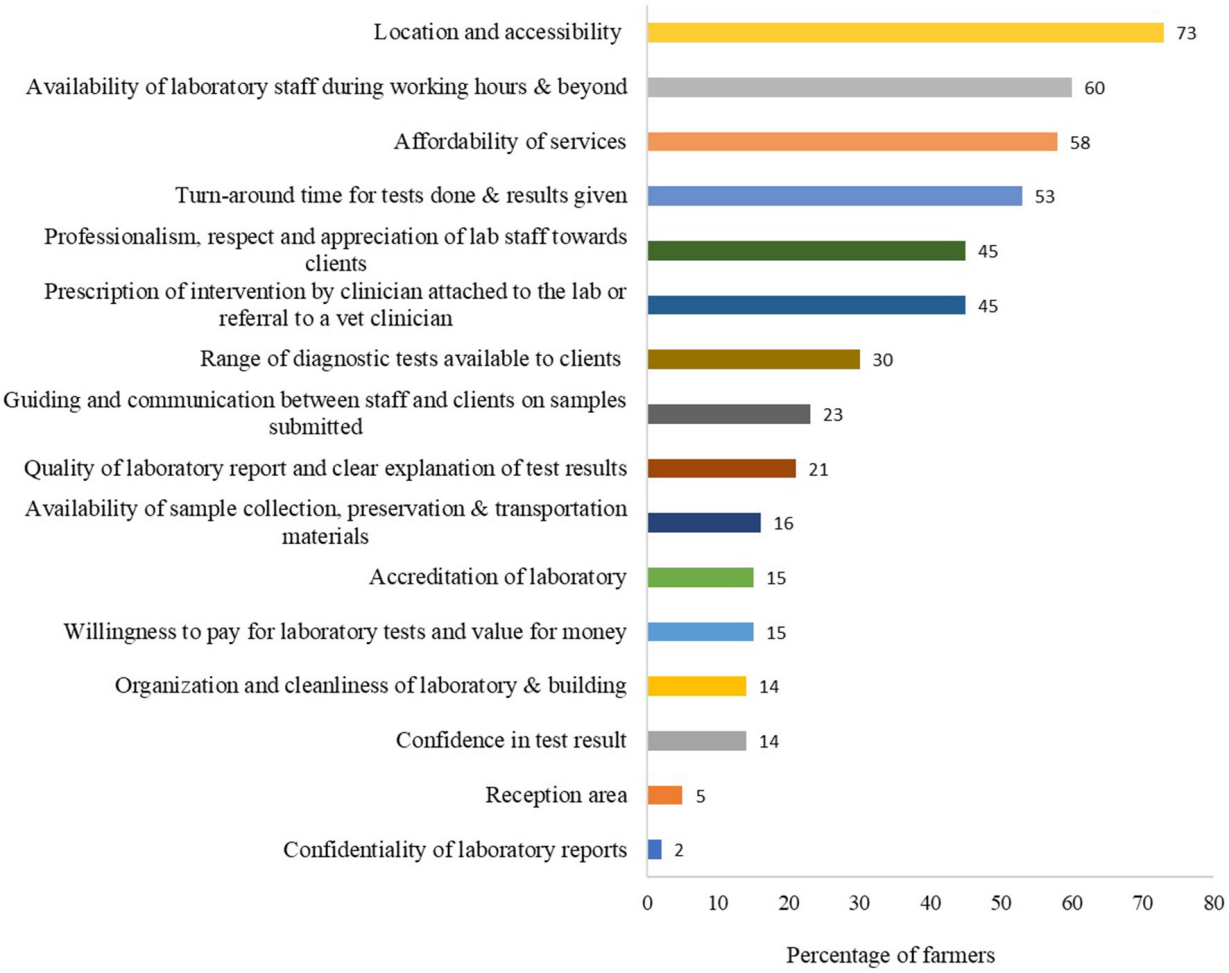
The Top ranked important factors that determine whether farmers would submit samples to a laboratory or not. Bars represent percent of farmers (*n* = 86) that selected each factor. This question had multiple responses.

Although up to 62% (53/86) of the farmers had never submitted samples to the laboratory, 21% (18/86) submitted samples either 1–2 or 3–6 times in past 6 months. Like the animal health workers, farmers also mainly submitted samples obtained from cattle, goats, and chicken (Supplementary Table 5 of Supplementary Data Sheet 1). Farmers were asked the factors that influence their decision to submit samples to a laboratory and to rank the factors in order of importance. The top four factors included location and accessibility (proximity of laboratory to farm premises) (63/86, 73%), availability of laboratory staff during working hours and beyond (52/86, 60%), affordability of the service (50/86, 58%), and sample turn-around time (46/86, 53%) (Figure 8). Other factors are shown in Figure 8 and Supplementary Table 8 of Supplementary Data Sheet 1.

The top five most important factors reported by both farmers and animal health workers were availability of laboratory staff (95/143, 66%), location and accessibility (91/143, 64%), affordability of services (68/143, 48%), professionalism, respect and appreciation of laboratory staff toward clients (67/143, 47%), and turn-around time for tests (66/143, 46%) (Supplementary Table 8 of Supplementary Data Sheet 1).

### Solutions to the Challenges Affecting Veterinary Diagnostic Laboratories

**Figure 9:**
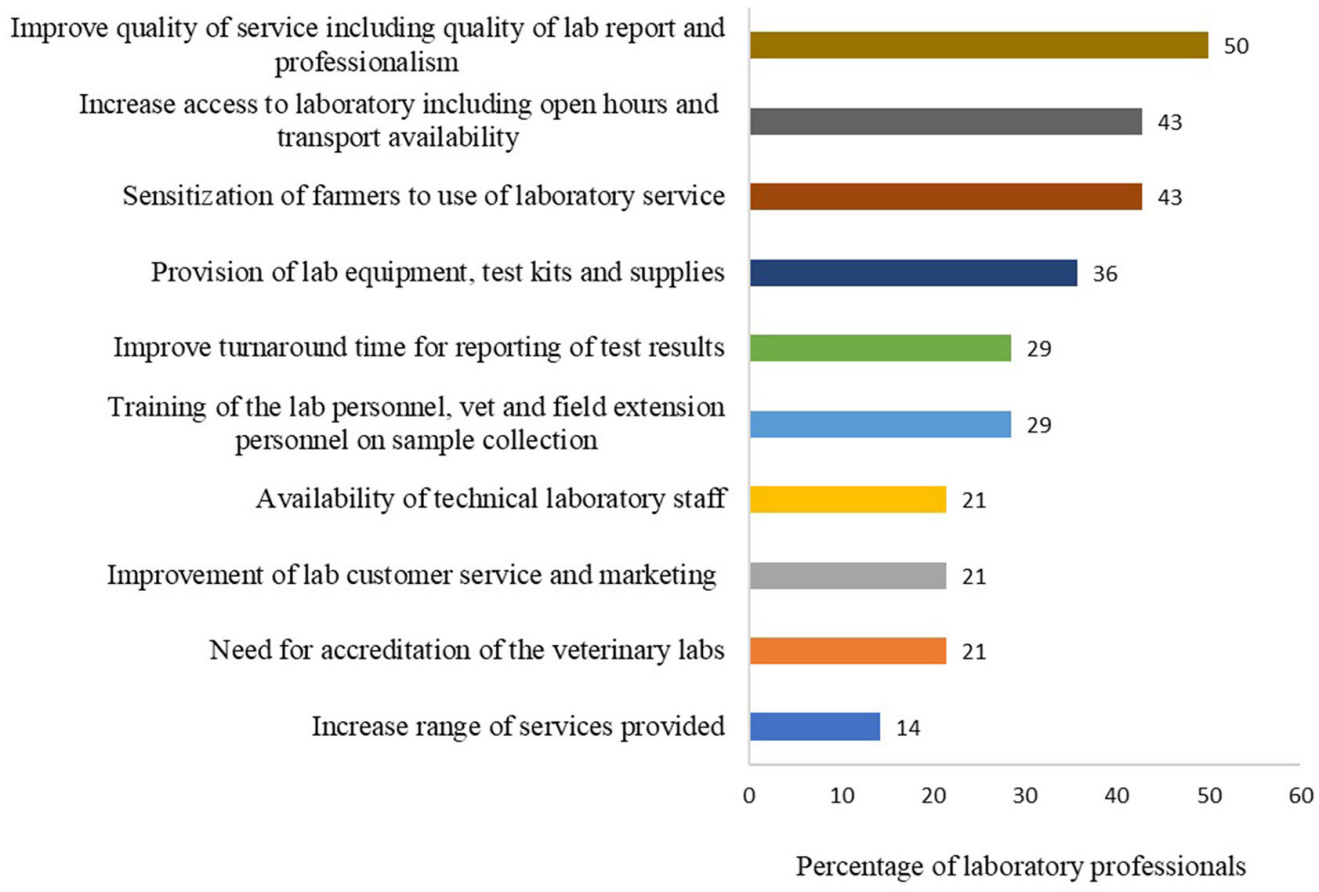
Key aspects that the laboratory needs to improve to attract more clients and improve client satisfaction. Bars represent percent of laboratory technicians (*n* = 14) that provided each solution. This question had multiple responses.

To understand possible ways of improving veterinary diagnostic services, the laboratory personnel were requested to list key aspects that they think need to be improved to attract more clients and improve client satisfaction. The top five proposed solutions included: improve quality of services (quality of result report and professionalism) (7/14), sensitization of farmers to promote use of laboratories for disease diagnosis (6/14), increase access to the laboratory (6/14), provision of laboratory equipment, test kits and supplies to veterinary laboratories (5/14), training (laboratory staff and vets on sample collection) (4/14) and improve turn-around time for test results (4/14) (Figure 9). Others included accreditation of the veterinary laboratories, improved laboratory customer care and marketing, availability of technical laboratory staff, and increased range of diagnostic services provided by the laboratories (Supplementary Table 9 of Supplementary Data Sheet 1).

One of the three themes identified from the key informant data was “solutions suggested by key informants in the veterinary sector.” The key informants were asked to give their opinion on how pre-existing and emerging veterinary diagnostic laboratories can be strengthened to provide sustainable diagnostic services across the livestock and poultry value chains. Their response generated a variety of recommendations for improvements of current and emerging veterinary diagnostic laboratories. Three codes emerged from the responses namely, education and training (code 1), funding and investment (code 2), and by-laws and policies (code 3). Code 1: Education and training, included the education of farmers on the benefits of performing laboratory diagnostics on animal health and welfare, on farmer income and farmer health, the sensitization of farmers on the dangers of self-treatment without a proper diagnosis, sensitizing farmers on benefits of laboratory diagnostics and the reasons for paying for services. Code 2: funding and investment, included the funds to run laboratory business on daily basis for example, to maintain power and cold chain, encourage farmers to pay for services to ensure sustainability, support farmers to get better value for their animal produce so that they can afford to pay for diagnostic services, reduce dependence on donor funding and find alternative sources of funding for laboratory diagnostic services to promote sustainability and improve pay for laboratory personnel to encourage more to enroll in laboratory training courses to become laboratory professionals. Lastly, code 3: by-laws and policies, included the regulation to restrict access to drugs to only qualified professionals. The most frequently referenced code in this theme was, “education and training.” The direct quotations from the key informants are shown in Box 3.

Box 3 **Solutions and recommendations suggested by key informants for improvements of current and emerging veterinary diagnostic laboratories in Uganda.**

**Table:** 

**Codes 1. Education and training**	**Code 2. Funding and investment**	**Code 3. By-laws and policies**
***KII:01**—*“*Demonstrating how useful the diagnostic approach is, showing its benefits. For example, A cow with subclinical mastitis giving 12 L after treatment, the cow gives another 4 L, such a farmer will never stop*.” ***KII:02**—*“*There should be a collaboration between laboratories and drug manufacturers such that on the drug bottle if they could write that before you use this drug, make sure your animal is tested so that farmers can be able to demand*.” ***KII:05**—*“*We need sensitization of farmers to avoid self-treatment*.” ***KII:06**—*“*Awareness campaigns, explain cost reduction, animal welfare issues, opportunities to access better markets, their own health impact on consuming animals on treatment*.”	***KII:01**—*“*Someone complained that the laboratory was using up a lot of power, so they switch it off and we could not maintain the cold chain because of lack of power, sometimes the problem remains over weekend and long periods of time*.” ***KII:02**—*“*Farmers have an attitude regarding government services which are given free of charge, hence demanding user fee from a farmer would be a problem, but anything given for free cannot be sustainable*.” ***KII:08**—*“*The prices of the commodities produced on livestock farms are quite discouraging so if they are not making good money from it, farmers cannot pay good price*.” ***KII:06**—*“*The funding support is definitely quite inadequate and depending mainly on donors like FAO and well-wishers*.” ***KII:08**—*“*The training of the personnel is not encouraging; the employment opportunities do not encourage people to invest in training to support these services in the laboratory field*.”	***Kll:04**—*“*We should record our animals to know what we have in the country such as a national record*.” ***KII:06**—*“*Currently, people in regulation need to have laboratories accredited such that services can be trusted by everybody, but we don't have*.” ***Kll:07**—*“*Currently, we do not have the veterinary laboratory diagnostic policy that governs the country. This leaves a lot of dilemma, we need standards so all service providers in terms of diagnostics should conform to a certain standard. We should do annual monitoring, and check they are all registered*.” ***Kll:08**—*“*Government policy that would require results from diagnostic services for certification of some products from livestock and food that is on the market*.”

*KII, Key informant interview*.

### Level of Satisfaction of Animal Health Workers and Farmers With Veterinary Diagnostic Services

The animal health workers were asked to rate their level of satisfaction with various aspects of veterinary diagnostic services including laboratory facilities, laboratory staff, and the tests carried out plus post-test services. Regarding laboratory facilities, most of the animal health workers expressed that they were either slightly or very satisfied with the location, organization, cleanliness, and reception area of the laboratories in their area (Supplementary Table 10 of Supplementary Data Sheet 1). A half of the animal health workers were very satisfied with the demeanor of staff (32/57, 56%) and appreciation of clients (30/57, 53%) (Supplementary Table 11 of Supplementary Data Sheet 1). Regarding level of satisfaction with the tests carried out by the veterinary diagnostic laboratories, animal health workers were mainly slightly or very satisfied with the various aspects of tests carried out including the diagnostic tests performed, affordability of laboratory tests, turnaround time, confidence in test results, quality of laboratory report, report confidentiality and value for money (Supplementary Table 12 of Supplementary Data Sheet 1), and the post-test services (Supplementary Table 13 of Supplementary Data Sheet 1) of the laboratories.

The farmers were also asked to rate their level of satisfaction with various aspects of veterinary diagnostic services including laboratory facilities, laboratory staff, the tests carried out and post-test services. Regarding laboratory facilities, majority of the farmers were neither satisfied nor dissatisfied or not sure about their level of satisfaction (Supplementary Table 10 of Supplementary Data Sheet 1). Similarly, most farmers were not sure, or neither satisfied nor dissatisfied with laboratory staff (Supplementary Table 11 of Supplementary Data Sheet 1), the tests carried out by the veterinary diagnostic laboratories, (Supplementary Table 12 of Supplementary Data Sheet 1), and the post-test services of the laboratories (Supplementary Table 13 of Supplementary Data Sheet 1).

## Discussion

This study aimed to identify the priority livestock diseases for which farmers and animal health professionals require veterinary diagnostics and the key clients for veterinary laboratory diagnostic services, assess the perceptions and opinions of key stakeholders on veterinary diagnostic services, identify the factors that influence diagnostic laboratory service utilization, and to propose relevant strategies that can be adopted by existing and emerging veterinary laboratories to improve animal disease diagnostic service delivery in Uganda. To achieve the above objectives, the key stakeholders in veterinary diagnostic service provision, utilization and regulations were targeted and these included animal health workers, laboratory technicians/technologists, farmers, and key informants knowledgeable with veterinary service delivery.

### Key Clients for Veterinary Laboratory Diagnostic Services

The results of the key informant interviews revealed that the key clients for veterinary laboratory diagnostic services were farmers, veterinarians, investors and researchers, co-operative societies, non-governmental organizations (NGOs), community-based organizations (CBOs), FAO, and pharmaceutical companies. Although farmers often seek diagnostic services directly from animal health professionals, they also seek diagnostic services through their cooperatives or associations. This is common with cattle farmers, especially dairy and beef cooperatives who tend to be well-organized with clear leadership structure and strategic goals (17). Veterinary diagnostic laboratories could leverage on the cooperative structure to create awareness of their services and to explain the value farmers can derive by using diagnostic services. Investors are also clients for veterinary laboratories and are mainly involved in the poultry business. The poultry sector is growing rapidly in the Kampala metropolitan area and emerging towns across the country (18). Commercial layers, broilers and breeder farms are a major client to laboratories in the Kampala metropolitan area due to their proximity to the laboratories. Another reported client for laboratory services that supports farmers is community-based organizations. The CBOs are more entrenched particularly in Karamoja region where livestock is the most important heritage and source of livelihood. However, the region is vulnerable to climate extremes and livestock disease outbreaks which makes the Karamoja region more prone to famine and extreme poverty (19). Although the FAO was mentioned as a client for veterinary laboratory diagnostics, it plays an indirect role; FAO is a development partner to the Government of Uganda, it engages mainly the national laboratory (NADDEC) in animal disease outbreak investigations. Pharmaceutical companies also play an indirect role; have outreach programs on promotions of veterinary drugs and laboratory supplies and client support through extension. Therefore, veterinary pharmaceutical companies have a big stake and interest as partners for veterinary laboratories since accurate diagnosis enhances rational use of drugs and reduces the risk of emergence of resistance against drugs.

### Priority Livestock Diseases for Which Diagnostic Services Are Needed

Findings from the survey of laboratory personnel revealed that the diseases most tested for were theileriosis (East coast fever), brucellosis, helminthiasis, anaplasmosis, colibacillosis, coccidiosis and babesiosis. Additionally, findings from the key informant interviews also reported the common diseases and conditions for which diagnostic services were needed. The reported cattle diseases included hemoparasites (tickborne diseases and trypanosomosis), viral diseases (FMD, LSD, RVF, papillomatosis), bacteria diseases (brucellosis, anthrax, leptospirosis, paratuberculosis), parasites (helminths), reproductive diseases or conditions (abortion, fetal deaths) and mastitis. The current study findings agree with the reports on the commonly diagnosed cattle diseases by Byaruhanga et al. (1) and Nakayima et al. (10). Byaruhanga et al. (1) conducted a retrospective study of laboratory records and identified the following as major cattle diseases; East coast fever, helminthiasis, mastitis, brucellosis and rabies. Nakayima et al. (10) surveyed animal health workers and identified the following as key cattle diseases; East coast fever, anaplasmosis, babesiosis, heart water, trypanosomosis, brucellosis, CBPP, mastitis; tuberculosis, and FMD. Regarding pigs, the key informants reported African swine fever (ASF) and porcine erysipelas, and for poultry, no specific reference was made to any disease. However, laboratory personnel reported coccidiosis in the current study, and this was also reported by Byaruhanga et al. (1).

On the other hand, the most frequently reported diseases the laboratories were unable to test included avian influenza, CBPP, FMD, PPR, Rift valley fever, Swine fever, brucellosis, and tick-borne diseases such as heart water. Some of these diseases were also reported among the most tested diseases including FMD, RVF, brucellosis and tick-borne diseases. Even though the reasons why the laboratories were unable to test for these diseases were not investigated in the current study, this finding suggests that the demand for diagnostic testing of these diseases (FMD, RVF, brucellosis, and tick-borne diseases) may be high, but provision is inadequate. Therefore, improving the provision of diagnostic services for these diseases as well as the other diseases previously mentioned above may contribute to veterinary diagnostic service delivery.

### Perceptions and Opinions of Key Stakeholders on Veterinary Diagnostic Services and the Factors That Influence Laboratory Service Utilization

The most common tests requested by clients as reported by laboratory technologists/technicians included microbial culture and antimicrobial sensitivity tests, serology, blood smear, post-mortem examination, and complete blood count. While the most common diagnostic tests requested by clients, but laboratories were unable to provide included rapid tests for CBPP, FMD, NDV and mycoplasma infection, toxicological tests especially acaricide analysis, general gram staining, culture and antimicrobial sensitivity test, serology, and complete blood count. Some of the diagnostic tests that laboratories were unable to provide were also reported among the most requested tests including, culture and antimicrobial sensitivity test, serology, and complete blood count. The reasons why the laboratories were unable to provide the tests were not investigated in the current study. The findings nevertheless also suggest that the demand for these diagnostic tests (culture and antimicrobial sensitivity test, serology, and complete blood count) may be high, but provision is inadequate. Subsequently, there is a need to improve provision of these diagnostic tests as well as the provision of other tests mentioned above at the laboratory level.

The current study findings suggest there is low capacity of veterinary laboratories to perform the requested diagnostic tests. Although microbial culture and antimicrobial sensitivity testing was the most common test requested by clients and reported as one of the tests that laboratories were unable to provide, only a few laboratories have the capacity to perform microbial culture. The regional veterinary laboratory in Mbarara district has the capacity to carryout microbial culture but is constrained by lack of budgetary support and inadequate laboratory supplies. The only participating laboratories that reported conducting microbial culture and antimicrobial sensitivity testing in this study were the microbiology laboratories at COVAB and NADDEC. Therefore, to promote antimicrobial stewardship, it's critical that regional veterinary laboratories are strengthened with the necessary equipment, biosafety containments, and human resource to be able to carry out culture and antimicrobial sensitivity testing. The availability and use of culture and antimicrobial sensitivity testing will help promote prudent antimicrobial use and prescription by animal health workers, thereby minimizing the risk of emergence of antimicrobial resistance.

Serology was the second most common requested test and was also reported as one of the tests that laboratories were unable to provide. It should be noted that only 2 participating laboratories reported to have an ELISA reader, suggesting that most of the reporting on serological testing was likely *Brucella* spp. screening using Rose Bengal test (RBT) as opposed to ELISA. Of the participating laboratories only RTC laboratory and NADDEC laboratory perform routine ELISA testing for vaccination monitoring and disease surveillance, respectively. Laboratories offering specialized serological tests for routine diagnosis and vaccine monitoring may benefit from expanding the scope of serological services offered to include endemic diseases for which laboratory diagnostic testing is needed such as FMD (20), PPR (21), African swine fever (ASF) (22), CBPP (23), and CCPP (24) among others. Expanding the diagnostic capacity of other laboratories will help to supplement NADDEC and improve the country's capacity to respond to sero-diagnostic needs.

Complete blood count (CBC) was also reported as one of the tests that laboratories were unable to provide yet was in demand. To address this need, veterinary laboratories need to consider introducing traditional CBC techniques but also evaluate the market feasibility of introducing automated hemo-analyzers. Considering the high initial cost of investment, automated hemo-analyzers could be introduced especially in large or regional laboratories. For other tests that laboratories were unable to provide such as toxicological tests, the laboratories should target clients who intend to carryout residue analysis for animal product sanitary certification.

This study found contrasting responses between farmers and animal health workers on the level of satisfaction of clients with veterinary laboratory diagnostic services. Whereas, animal health workers were slightly, or very satisfied, farmers were neither satisfied nor dissatisfied or not sure with the various aspects of veterinary diagnostic services including laboratory facilities (location, organization, cleanliness, and reception area of the laboratories), laboratory staff (demeanor of staff, appreciation of clients), and tests carried out (diagnostic tests performed, affordability of laboratory tests, turnaround time, confidence in test results, quality of laboratory report, report confidentiality, and value for money). These findings could be explained by the fact that sample submission to veterinary laboratories is mostly performed by animal health workers. Animal health workers interact more frequently with veterinary laboratories thus putting them in a better position to make fair assessment regarding laboratory services than farmers. The fact that animal health workers showed some level of satisfaction with veterinary laboratory services is a positive indicator for the laboratory service providers. However, the findings suggest there is a need to engage farmers to understand the value of laboratories in relation to disease diagnosis, so that farmers can fully support animal health workers to make laboratory diagnosis a priority before medication.

The perceptions of animal health workers, farmers and laboratory technologists and technicians on the most important factors that determine whether clients will submit samples to a laboratory were assessed. Both animal health workers and farmers indicated that the most important factors that determine whether they will submit samples to veterinary laboratories were sample test results turn-around time and location and accessibility (proximity of laboratory to farm premises). The findings in this study agree with a European study that investigated the opinions of veterinary practitioners in Northern Ireland (25). The study of Irish veterinary practitioners also found that sample turn-around time and professionalism (personal attributes of laboratory personnel) affected clients' satisfaction and positively influenced their decision to submit samples again to the laboratory (25). Veterinary laboratories in Uganda need to evaluate sample turn-around time and improve efficiency with which samples are analyzed and ensure laboratory reports are issued in a timely manner. A short turn-around time allows animal health workers to use the result for clinical intervention, save animal lives and prevent loses. It is also important for veterinary laboratories to communicate with their clients about the samples received and the results in a timely fashion. Communication is valued by clients and such interactions help to build positive relationships between the two parties while creating opportunities for the laboratory personnel to explain the results to the sample submitter. In Northern Ireland, Rubinson and Epperson (25) found that communication with pathologists was considered extremely important because it's a sign that the pathologist is interested and cares about the case.

Location and accessibility of veterinary laboratories was reported by both animal health workers and farmers as an important factor that influences their decision to submit samples to a laboratory. The proximity of a laboratory to those submitting the samples, whether it is animal health workers or farmers can play a big role; a longer distance or difficult access to the laboratory is likely to discourage sample submissions. Strategies aimed at increasing availability of laboratory facilities and improving access to laboratories are likely to contribute to increased sample submission to veterinary laboratories. To remain attractive to farmers, veterinary laboratories should also strive to make their services affordable. Most farmers ranked affordability of services among the most important determinants of sample submission. Equally, for veterinary laboratories to attract animal health workers to submit samples, they should continuously carryout market analysis to know the diagnostic needs of the clinicians and strive to improve the range of diagnostic tests offered. Whereas, laboratory accreditation was ranked least, it is important that veterinary laboratories develop quality management systems and aim to get accredited, in alignment with OIE quality standards for conducting tests for animal diseases (26). Accreditation gives credibility to the laboratory and permits such laboratories to offer services for regulatory certifications.

There were similarities in the challenges to providing diagnostic services reported by laboratory personnel and the key informant interview respondents. Both groups reported that the most frequent challenges to providing diagnostic services were poor equipment or lack of relevant equipment, insufficient or lack of supplies and reagents, high cost of reagents, inadequate or lack of laboratory staff to perform tests, and inadequate training of laboratory staff. A previous study that surveyed animal health workers reported similar challenges including inadequate laboratory space, lack of trained laboratory personnel, lack of equipment, lack of funds to purchase supplies, lack of motivation of veterinary staff to perform laboratory diagnosis, farmers not motivated to support laboratory diagnosis, and lack of required tests (10).

The lack of laboratory equipment, laboratory supplies and human capacity to perform tests were mentioned in all the study districts. It should be noted that at the district level, veterinary diagnostic services are not fully supported with recurrent budgets at both central and local governments. Considering that veterinary laboratories are mainly government owned, there is need for both central and local governments to take full responsibility for renovating, equipping, and employing laboratory staff at both district and regional/national laboratories. To encourage financial independence and promote sustainability, the government laboratories should be allowed to charge modest cost-recovery fees for diagnostic services offered. This is the strategy currently used by laboratories in Makerere University and the private laboratories established with support from Zoetis under the ALPHA initiative. Other strategies that may help achieve sustainability include investment in improving laboratory environments, purchase of laboratory equipment and supplies, and appropriate payment of laboratory human resources.

### Proposed Solutions That Can Be Adopted by Existing and New Veterinary Laboratories to Improve Diagnostic Laboratory Service Delivery

Laboratory technologists/technicians and key informants proposed ways veterinary diagnostic services can attract more clients and improve client satisfaction, including education of farmers on the benefits of performing laboratory diagnostics (on animal health and welfare, and on farmer income and health), and provision of laboratory equipment, test kits, and supplies to veterinary laboratories. The key informants also proposed ways veterinary diagnostic services can attract more clients and improve client satisfaction, including sensitization of farmers on dangers of self-treatment without proper diagnosis, encouraging farmers to pay for services to ensure sustainability, supporting farmers to get better value for their animal produce so can afford to pay for diagnostic services, reduce dependence on donor funding and find alternative sources of funding for laboratory diagnostic services to promote sustainability, improve pay for laboratory personnel to encourage more to enroll in laboratory training courses to become laboratory professionals.

The current study agrees with Nakayima et al. (10) that veterinary laboratories in Uganda struggle to sustain their operations and strategies to reverse this trend are needed. Almost all the stakeholders that participated in the current study proposed increased awareness on the value of diagnostic laboratories and investment in equipping veterinary laboratories to offer better services. Increasingly, commercial farmers in the poultry sector are recognizing the above reality. For example, for a poultry breeder to compete favorably, they must assure their client that the parent stocks and day-old chicks are healthy. As a result, local poultry companies in Uganda now routinely submit samples to laboratories for poultry serology. Similarly, upcountry laboratories established under the Zoetis ALPHA initiative are now operational and are being utilized by animal health workers in addition to the district laboratories because of the increased awareness among livestock farmers/cooperatives about the value of the laboratories coupled with proximity of the laboratory to their farms. This allows for veterinary laboratory staff to explain and demonstrate the value of testing before treatment. One of the key informants stated it well “*Demonstrating how useful the diagnostic approach is, showing its benefits. For example, A cow with subclinical mastitis giving 12 liters after treatment, the cow gives another 4 liters, such a farmer will never stop*.” Therefore, it is important for all veterinary laboratories in the country to reach out to animal health professionals and farmers, promote the diagnostic solutions to their clients, demonstrate the value added by diagnostics to the client's business and use the revenues generated to improve laboratory facilities, supplies, and other operational costs if they are to run sustainably.

For the district and regional government laboratories, there is a need for more government commitment to provide budget and other support for operationalizing diagnostic laboratories. There should be a deliberate effort to expand the human resource at district and regional veterinary departments to include positions for laboratory technicians and technologists. With recurrent financial support, the districts, and regional laboratories will help to reduce pressure on the national animal diagnostic laboratory (NADDEC) in both active and passive surveillance as well as outbreak investigations. Part of the investment in the laboratories should also cover installation of power backup systems to avoid service interruptions due to power outage. It is worth noting that the proximity of district veterinary laboratories to farmers is an incentive for sample submission and evidence-based drug prescription. Therefore, government support to increase access to laboratories will contribute toward veterinary diagnostic service delivery and to efforts that promote appropriate antimicrobial drug use.

Clearly veterinary diagnostic laboratories would benefit from the support of the government, farmers and the livestock industry. Support for veterinary diagnostic laboratories in Uganda will improve the quality of veterinary services delivered to livestock farming communities, and as a consequence the high burden of livestock diseases in farms will be mitigated. These actions will create positive influences on the economy of farming communities, which currently are hampered by the rampant livestock diseases, while numerous investment opportunities would also be identified along the livestock value chains and hence boosting the general economy of the nation.

Overall, the management of the various veterinary laboratories need to develop quality management system that must be implemented, reviewed, and improved. This will ensure that human resources in the laboratory are well-trained and competent and provide quality customer care, laboratory equipment are maintained and calibrated, scope of tests offered is progressively increased to respond to market demands, and that laboratories develop strategic plans and work toward accreditation.

## Conclusions and recommendations

This study has identified the priority livestock diseases for which diagnostic services were needed, and revealed that some common endemic viral, bacterial, and tick-borne diseases are not tested due to lack of test capacity at laboratories. The study findings suggest that there is a need to improve provision of culture and antimicrobial sensitivity testing, serology and complete blood count testing because current provision of these tests is inadequate yet there is demand. To improve sample submission rate, strategies must be developed at the government and local laboratory levels to ensure that the most important determinants of sample submission are addressed appropriately, including increasing availability of laboratory facilities, improving access to laboratories, reducing sample test results turn-around time, increasing range of diagnostic tests available to clients, making diagnostic services affordable, improving professionalism of laboratory staff, respect and appreciation of laboratory staff toward clients, and quality of laboratory reports. Study findings also revealed that veterinary diagnostic services in Uganda are financially constrained, and improved funding and budgeting within the services provided by government and private sector may help address the reported challenges such as poor equipment or lack of relevant equipment, insufficient or lack of supplies and reagents, high cost of reagents, and inadequate or lack of laboratory staff to perform tests. Finally, the education of farmers and animal health workers on the value and benefits of performing laboratory diagnostics may help contribute to increase in sample submission and subsequent demand for diagnostic services. And the improvement in quality management systems at the laboratory level through attainment of accreditation may increase the confidence of clients in laboratory results and contribute to overall strengthening of the veterinary laboratory diagnostic services in the country.

## Data Availability Statement

The raw data supporting the conclusions of this article will be made available by the authors, without undue reservation.

## Ethics Statement

The studies involving human participants were reviewed and approved by Makerere University, School of Veterinary Medicine and Animal Resources Institutional Animal care and Use Committee (Protocol number: SVAR-IACUC/46/2020) and the Uganda National Council for Science and Technology (Ref number: A84ES). The participants provided their written informed consent to participate in this study.

## Author Contributions

AE and PV conceived the study. AE, PV, JB, IE, RA, and AC developed study protocol—including data collection strategy and instruments. PV, JB, AE, and IE were involved in data collection activities. SW, NG, IE, AE, and PV conducted data analysis and interpretation. AE, SW, NG, JB, and PV were involved in preparation of the manuscript. AE, PV, SW, NG, JB, IE, RA, EM, GV, and AC contributed to the final version of the manuscript. All authors contributed to the article and approved the submitted version.

## Funding

This study was supported by the African Livestock Productivity and Health Advancement (ALPHA) Initiative, co-funded by the Bill and Melinda Gates Foundation (BMGF) and Zoetis. Funding from Zoetis was an unrestricted grant. BMGF Grant number: OPP1165393.

## Author Disclaimer

The views and opinions expressed in this article are those of the authors and do not necessarily reflect the policy or position of any affiliated agency or institution of the authors.

## Conflict of Interest

EM and GV are employed by Zoetis and contributed to conception and report writing. The remaining authors declare that the research was conducted in the absence of any commercial or financial relationships that could be construed as a potential conflict of interest.

## Publisher's Note

All claims expressed in this article are solely those of the authors and do not necessarily represent those of their affiliated organizations, or those of the publisher, the editors and the reviewers. Any product that may be evaluated in this article, or claim that may be made by its manufacturer, is not guaranteed or endorsed by the publisher.
