# Supplementary material for: A Survey of Priority Livestock Diseases and Laboratory Diagnostic Needs of Animal Health Professionals and Farmers in Uganda
Source: Front Vet Sci. 2021 Sep 23;8:721800. doi: 10.3389/fvets.2021.721800 (PMC8494769; doi:10.3389/fvets.2021.721800)
Supplement: Supplementary file 1 [file Data_Sheet_1.pdf]

Supplementary Table 1: Number of livestock by district and type based on 2008 Uganda Livestock Census

Region	District	Cattle	Goats	Sheep	Pigs	Chicken	Ducks	Turkey
**Central**	Kalangala	5,814	5,762	0	6,547	58,088	8,080	157
	Kampala	31,614	64,072	8,790	38,306	1,053,031	28,148	5,675
	Kiboga	365,154	105,250	26,270	49,595	428,601	4,582	883
	Luwero	79,787	68,527	13,275	59,040	464,943	7,032	1,398
	Masaka	224,600	244,706	28,652	236,148	1,108,363	58,723	16,223
	Mpigi	216,621	102,828	23,221	108,082	600,950	10,456	1,143
	Mubende	208,535	139,400	31,094	98,487	536,342	12,525	1,614
	Mukono	155,820	206,704	30,808	181,846	1,551,702	49,517	5,558
	Nakasongola	222,185	87,823	6,839	35,283	287,834	6,316	550
	Rakai	279,594	163,806	18,158	102,870	503,623	15,399	1,097
	Ssembabule	177,473	113,204	14,219	35,399	194,462	10,011	2,528
	Kayunga	88,814	82,701	7,707	38,067	327,603	14,327	760
	Wakiso	114,769	132,964	27,542	199,962	2,783,509	33,350	4,852
	Lyantonde	68,572	58,642	5,590	7,770	73,588	2,392	259
	Mityana	75,767	51,029	18,000	80,346	364,398	8,449	1,786
	Nakaseke	160,737	48,634	9,439	29,706	193,392	1,995	245
**Sub Total**		**2,475,856**	**1,676,052**	**269,604**	**1,307,454**	**10,530,429**	**271,302**	**44,728**
**Eastern**	Bugiri	118,427	220,778	14,280	65,453	943,073	74,332	6,229
	Busia	26,787	73,565	2,908	14,203	391,312	13,041	3,777
	Iganga	125,307	169,915	5,064	27,684	904,493	13,469	6,550
	Jinja	40,247	71,893	1,691	26,856	524,159	10,456	2,463
	Kamuli	211,815	219,194	6,540	55,239	724,489	15,538	2,421
	Kapchorwa	95,564	75,073	9,852	8,070	285,543	2,898	427
	Katakwi	136,966	104,932	25,511	19,381	286,229	4,902	3,423
	Kumi	220,055	168,887	30,994	67,650	549,135	9,936	20,360
	Mbale	63,826	96,617	5,108	23,315	459,868	13,100	26,162
	Pallisa	136,225	149,003	20,488	25,302	440,035	20,748	27,928
	Soroti	271,634	236,839	53,010	75,449	808,290	23,910	19,677
	Tororo	119,587	154,058	13,086	45,256	591,552	24,624	33,535
	Kaberamaido	76,109	97,516	33,566	31,607	367,924	13,146	1,850
	Mayuge	85,523	135,669	8,010	18,345	607,880	59,740	13,677
	Sironko	92,562	79,141	9,806	32,733	391,125	8,496	19,769
	Amuria	171,375	113,110	35,942	41,318	545,388	5,703	4,670
	Budaka	40,231	51,942	3,987	5,043	172,627	6,933	8,940
	Bududa	50,809	25,885	4,012	21,386	205,703	2,153	6,061
	Bukedea	86,141	54,810	10,013	23,264	215,251	4,400	5,596
	Bukwo	23,360	23,312	2,137	1,657	94,993	1,761	126

	Butaleja	77,247	71,609	9,732	4,497	251,946	18,524	10,397
	Kaliro	65,364	56,090	2,144	14,775	188,942	3,694	2,298
	Manafwa	76,602	79,928	4,795	38,905	444,266	7,405	8,658
	Namutumba	76,704	70,212	6,691	12,287	301,875	7,995	3,030
**Sub Total**		**2,488,467**	**2,599,978**	**319,367**	**699,675**	**10,696,098**	**366,904**	**238,024**
**Northern**	Adjumani	131,282	26,030	26,030	7,449	391,626	26,267	971
	Apac	225,088	279,649	45,967	28,442	939,652	34,899	4,043
	Arua	117,157	273,012	45,922	22,927	588,824	21,468	1,402
	Gulu	40,130	65,301	4,289	26,569	299,830	62,358	5,211
	Kitgum	38,457	54,815	11,509	38,444	139,286	31,949	1,234
	Kotido	694,247	535,138	555,688	1,318	219,598	12,737	3,863
	Lira	159,533	161,711	12,749	28,631	1,116,903	30,927	4,927
	Moroto	352,867	380,172	307,028	5,534	260,997	18,834	3,075
	Moyo	103,873	190,341	37,742	9,034	373,086	15,808	776
	Nebbi	101,952	302,576	46,084	19,895	583,704	34,727	1,304
	Nakapiripirit	674,746	547,365	389,676	322	314,308	15,653	1,095
	Pader	57,087	57,807	6,298	39,430	150,317	43,197	1,144
	Yumbe	223,649	409,793	151,356	17,511	709,483	10,888	1,097
	Abim	13,635	37,229	8,381	17,354	61,330	3,373	2,213
	Amolatar	81,269	70,318	34,293	11,503	265,076	15,780	1,189
	Amuru	33,063	67,092	9,773	19,180	142,121	44,754	2,558
	Dokolo	58,902	71,815	16,361	13,602	291,027	14,777	623
	Kaabong	518,465	525,389	424,729	33,829	506,585	16,849	1,551
	Koboko	54,204	101,602	33,250	272	209,513	9,742	648
	Nyadri	123,640	286,929	67,543	29,222	793,213	32,534	2,137
	Oyam	118,603	172,052	19,347	28,350	650,758	21,918	2,606
**Sub Total**		**3,921,849**	**4,616,136**	**2,254,015**	**398,818**	**9,007,237**	**519,439**	**43,667**
**Western**	Bundibugyo	163,913	131,765	14,824	14,692	312,931	27,645	519
	Bushenyi	207,184	376,561	79,757	57,467	364,568	19,971	2,343
	Hoima	109,998	187,128	**25,593**	104,669	942,843	26,898	2,677
	Kabale	98,552	201,597	83,060	22,255	218,800	5,726	865
	Kabarole	67,115	155,264	13,510	40,781	352,530	8,990	1,742
	Kasese	97,243	227,518	24,890	85,812	752,800	45,036	4,694
	Kibaale	174,926	199,572	24,329	153,512	879,032	34,194	2,140
	Kisoro	28,083	96,815	39,554	10,171	111,347	1,481	128
	Masindi	213,402	233,423	24,943	87,616	1,007,182	39,362	1,843
	Mbarara	149,992	176,464	22,588	12,243	239,470	5,966	711
	Ntungamo	229,004	273,284	41,556	8,899	184,760	8,814	457
	Rukungiri	60,061	134,757	19,262	25,176	138,100	5,858	515
	Kamwenge	120,906	154,422	26,239	34,280	339,191	11,237	363

	Kanungu	31,120	105,498	12,849	22,900	196,564	8,701	427
	Kyenjojo	184,537	254,966	38,235	73,345	579,743	6,712	598
	Buliisa	34,801	43,326	3,884	849	99,932	18,542	115
	Ibanda	55,126	89,704	13,997	12,164	144,301	6,851	153
	Isingiro	180,345	221,491	30,298	7,552	203,564	13,905	1,370
	Kiruhura	342,315	188,686	28,017	3,967	142,459	4,719	235
**Sub Total**		**2,548,623**	**3,452,241**	**567,385**	**778,350**	**7,210,117**	**300,608**	**21,895**
**Uganda**		**11,434,795**	**12,344,407**	**3,410,371**	**3,184,297**	**37,443,881**	**1,458,253**	**348,314**

Source: Ministry of Agriculture, Animal Industry and Fisheries (MAAIF), and Uganda Bureau of Statistics (14).

Supplementary Table 2*:* Category of respondents and estimated number of respondents targeted per district before study implementation.

	Central			Southwest			
	Kampala	Wakiso	Mukono	Nakaseke	Mbarara	Ssembabule	**Total**
Laboratory technicians	5	2	1	1	2	1	12
Veterinary practitioners	4	4	4	4	4	4	24
Animal production	2	2	2	2	2	2	12
AHO	2	2	2	2	2	2	12
Commercial Cattle	0	2	4	8	10	8	32
Commercial Pig	2	10	10	0	2	2	26
Commercial poultry	4	10	10	2	4	0	30
Key informants	7	1	1	1	1	1	12
**Total**	26	33	34	20	27	20	**160**

AHO=Animal Husbandry Officer

Supplementary Table 3: Demographic characteristics and perceptions of laboratory technologists/technicians on veterinary diagnostic services.

Question	Variable	Frequency	Percentage
Laboratory description (n=16)	Academic (research/teaching) laboratory	7	44
	Government	6	38
	Private laboratory	1	6
	Public/non-government	2	12
Years worked in laboratory (n=16)	0-5 years	6	38
	6-10 years	5	31
	11-20 years	2	12
	Over 20 years	3	19
Clinicians appreciate laboratory Diagnosis (n=14)	I'm not sure	1	7
	Yes	13	93
	No	0	0
Farmers appreciate laboratory Diagnosis (n=14)	I’m not sure	2	14
	Yes	12	86
	No	0	0
Farmers willingness to pay for diagnostic services (n=14)	I'm not sure	2	14
	No	1	7
	Yes	11	79
Can Veterinary diagnostic laboratories run profitably (n=14)	I'm not sure	2	14
	No	1	7
	Yes	11	79
Have you ever conducted customers surveys to understand diagnostic need of clients (n=14)	I'm not sure	1	7
	Do not conduct marketing survey	7	50
	Conduct marketing survey	6	43
Attended continued professional training in the last 2 years (n=14)	No	4	29
	Yes	10	71
If no, reasons for non-attendance of professional training (n=4)	Training not available	3	75
	Organisational problems - both local government and MAAIF	1	25

Supplementary Table 4: Demographic characteristics and perceptions of animal health workers on veterinary diagnostic services.

Question	Variables	Frequency	Percentage
**Employment sector (n=57)**	Academic (research/teaching) lab	3	5
	Government employee	39	68
	Non-governmental organisation employee	5	9
	Private practice		
**Years of work practice experience (n=57)**	0-5 years	15	26
	11-20 years	21	37
	6-10 years	15	26
	Over 20 years	6	6
	Prefer not to say	0	0
**Type of current practice (n=57)**	Exotic practice (rabbits, parrots, tortoise, snakes etc.)	1	2
	Mixed practice (two or more of the practices listed above)	18	32
	Poultry practice (chickens, turkey etc.)	7	12
	Ruminants (cattle, sheep, goats etc.)	27	47
	Small animal practice (dogs, cats)	1	2
	Others	3	5
**Frequency of sample submission (n=57)**	1-2 times in six months	15	26
	1-3 times per month	6	11
	3-6 times in six months	16	28
	Over 3 times a month	12	21
	Never	8	14
	Don't know	0	0
**Animal species from which samples were submitted**	Cattle	42	74
	Chickens	17	30
	Goats	23	40
	Pigs	9	16
	Sheep	3	5
	Horse, Donkeys, Mules	0	0
	Ducks or geese	1	1
	Turkeys	1	1
	Others	1	1

Supplementary Table 5: Demographic characteristics and perceptions of farmers on veterinary diagnostic services.

Question	Variable	Frequency	Percentage
**Livestock production sector in which farmers are involved (n=86)**	Cattle farming	29	34
	Pig farming	24	28
	Poultry farming	32	37
	Others	1	1
**Type of animal species kept at present**	Cattle	45	52
	Chickens	45	52
	Goats	35	41
	Pigs	34	40
	Sheep	7	8
	Horses, donkeys, or mules	1	1
	Ducks or geese	1	1
	Turkeys	2	2
	Fish/ aquaculture	0	0
**Number of years of experience in farming (n=86)**	0-5 years	21	24
	6 - 10 years	25	29
	11- 20 years	15	17
	Over 20 years	24	28
	Prefer not to say	1	1
**Frequency of sample submission in the last 6 months (n=86)**	1-2 times in six months	18	21
	1-3 times per month	0	0
	3-6 times in six months	12	14
	Over 3 times a month	0	0
	Never	53	62
	Don't know	3	3
**Animal species whose sample were submitted mostly**	Cattle	20	23
	Chickens	11	13
	Goats	8	9
	Pigs	3	3
	Sheep	1	1
	Horses, Donkeys, Mules	0	0
	Ducks or geese	0	0
	Turkeys	0	0
	Others	0	0

Supplementary Table 6: Reported trainings attended by laboratory technologists/technicians (n = 10).

Training list
Administration and management
Audit management
Basic laboratory techniques
Bio-risk management
Biosafety and biosecurity principles.
Biosafety and Biosecurity training in basic microbiology techniques held at COVAB 2020
Equipment maintenance and calibration
Infectious disease diagnostics in disease surveillance
Laboratory management
Orientation training at NADDEC
Post-mortem of chicken by ZOETIS
Quality assurance
Rabies diagnostics (FAT; ELISA testing and rabies viral neutralisation test)
Research
Zoonotic diseases - samples handling to prevent animal to human disease transmission

Supplementary Table 7: The list of instruments/equipment most often used in the laboratories. The data shows instruments and proportion of laboratory technicians (n = 16) who mentioned each type of instrument/equipment.

Instrument	Frequency of reporting (n=16)	Percentage
Microscope	12	75
Fridge/Freezer	8	50
Pipettes	7	44
Incubator	7	44
Centrifuge	7	44
Autoclave	5	31
Biosafety cabinet	4	25
Weighing Balance	4	25
Computer	3	19
PH meter	3	19
Steamers/water bath	3	19
Bunsen burner	2	13
Differential tally counter	2	13
ELISA Reader	2	13
Glass slides	2	13
Hot air Oven	2	13
Microtome	2	13
PCR Machine	2	13
Water distiller	2	13
Automatic Shaker	1	6
Auto-tissue processors	1	6
Cooler box	1	6
Digital pregnancy scanner	1	6
Embedding assemblies	1	6
Gas cylinder.	1	6
Gel documentation machines	1	6
Haematocrit centrifuge	1	6
HB meter	1	6
Heat block	1	6
Histokinette	1	6
Hood	1	6
HPLC	1	6
Ice maker	1	6
Lyophiliser	1	6
McMaster slide	1	6
Microwave	1	6
Pipette tips	1	6
Plate Washer machine	1	6
PM kit	1	6

Instrument	Frequency of reporting (n=16)	Percentage
Refractometer	1	6
Sahli apparatus	1	6
Spreaders/inoculating loop	1	6
Staining rack	1	6
Tiles	1	6
Water purifiers	1	6

Supplementary Table 8: Factors that influence the decision of farmers (n = 86) and animal health workers (n= 57) to submit samples to the laboratory.

**Very important factors**	**All**	**Farmers**	**AHW***
	**n (%)**	**n (%)**	**n (%)**
Confidentiality of laboratory reports	5 (3)	2 (2)	3 (5)
Reception area (someone present and space to sit)	6 (4)	4 (5)	2 (4)
Willingness to pay for laboratory tests and value for money	21 (15)	13 (15)	8 (14)
Availability of sample collection, preservation and transportation materials	25 (17)	14 (16)	11 (19)
Accreditation (National or international) of laboratory	26 (18)	13 (15)	13 (23)
Confidence in test result	26 (18)	12 (14)	14 (25)
Organization and cleanliness of laboratory and building	27 (19)	12 (14)	15 (25)
Guiding and communication between staff and clients on samples submitted	35 (24)	20 (23)	15 (26)
Quality of laboratory report (completeness, comprehension etc) and clear explanation of test results	40 (28)	18 (21)	22 (39)
Prescription of intervention by clinician attached to the laboratory or referral to a veterinary clinician	51 (36)	39 (45)	12(21)
Range of diagnostic tests available to clients	55 (38)	26 (30)	29 (51)
Turn-around time for tests done and results given	66 (46)	46 (53)	20 (35)
Professionalism, respect and appreciation of laboratory staff towards clients	67 (47)	39 (45)	28 (49)
Affordability of services	68 (48)	50 (58)	18 (32)
Location and accessibility (proximity of laboratory to my farm/ home)	91 (64)	63 (73)	28 (49)
Availability of laboratory staff during working hours and beyond	95 (66)	52 (60)	43 (75)

*AHW = Animal Health Worker

Supplementary Table 9: Key aspects that require improvement in order to attract more clients and improve client satisfaction with veterinary diagnostic services, as reported by laboratory personnel (n = 14).

Themes of suggested solutions to challenges affecting veterinary laboratories	Frequency (n=14)	Percentage
Sensitization of farmers to use of laboratory service	6	43
Improve quality of service including quality result report and professionalism	7	50
Increase access to laboratory including open hours and transport availability	6	43
Provision of lab equipment, test kits and supplies	5	36
Training of the lab personnel, vet and field extension personnel on sample collection	4	29
Improve turnaround time for reporting of test results	4	29
Need for accreditation of the veterinary labs	3	21
Improvement of lab customer service and marketing	3	21
Availability of technical laboratory staff	3	21
Increase range of services provided	2	14
**Others**		
Subsidize costs/charges to make the service affordable	1	7
Power backup/standby generator to help in offering uninterrupted diagnostic services.	1	7
Lab management/administration	1	7
Referral system of samples	1	7
Need for labs to specialize (eliminate service duplication)	1	7
Improve biosafety (waste disposal procedures)	1	7
Improve lab information collection systems	1	7

Supplementary Table 10: Satisfaction of farmers (n = 86), animal health workers (n = 57) and all combined (n = 143) with the laboratory facilities (location, organisation, cleanliness, and laboratory reception area).

	Location			Organisation			Cleanliness			Reception area		
	All	Farmers	AHW	All	Farmers	AHW	All	Farmers	AHW	All	Farmers	AHW
**Rate**	n (%)	n (%)	n (%)	n (%)	n (%)	n (%)	n (%)	n (%)	n (%)	n (%)	n (%)	n (%)
I'm not sure	27 (19)	25(30)	2 (4)	29 (21)	27 (32)	2(4)	33 (24)	30 (36)	3 (5)	31 (23)	29 (35)	2 (4)
Neither satisfied nor dissatisfied	23 (16)	20 (24)	3 (5)	25 (18)	23 (27)	2 (4)	25 (18)	22 (27)	3 (5)	29 (21)	24 (29)	5 (9)
Slightly dissatisfied	4 (3)	3 (4)	1 (2)	6 (4)	1 (1)	5 (9)	0	0	0	1 (1)	0	1 (2)
Very dissatisfied	3 (2)	1 (1)	2 (4)	2 (1)	1 (1)	1 (2)	2 (1)	0	2 (4)	2 (1)	0	2 (4)
Slightly satisfied	40 (28)	9 (11)	31 (54)	31 (22)	8 (10)	23 (41)	30 (22)	10 (12)	20(36)	34 (25)	13 (16)	21 (39)
Very satisfied	44 (31)	26 (31)	18 (32)	47 (34)	24 (29)	23 (41)	49 (35)	21 (25)	28 (50)	40 (29)	17 (20)	23 (43)

Supplementary Table 11: Satisfaction of farmers (n = 86) and animal health professionals (n = 57) with the laboratory staff (staff availability, staff appearance, staff demeanour, staff appreciation to clients, and sample submission and processing turnaround time).

	Availability		Appearance		Demeanour		Appreciation of Clients		Turn-around time	
**Rate**	Farmers	AHW	Farmers	AHW	Farmers	AHW	Farmers	AHW	Farmers	AHW
	n (%)	n (%)	n (%)	n (%)	n (%)	n (%)	n (%)	n (%)	n (%)	n (%)
I'm not sure	27 (31)	2 (4)	27 (32)	2 (4)	27 (32)	2 (4)	27 (32)	2 (4)	24 (29)	2 (4)
Neither satisfied nor dissatisfied	23 (27)	3 (5)	23 (27)	2 (4)	22 (26)	4 (7)	23 (27)	3 (5)	22 (26)	6 (11)
Slightly dissatisfied		1 (2)		1 (2)	1 (1)	2 (4)	1 (1)	2 (4)	2 (2)	3 (5)
Very dissatisfied		2 (4)		2 (4)	1 (1)	1 (2)	1 (1)	1 (2)	1 (1)	1 (2)
Slightly satisfied	8 (9)	26 (46)	7 (8)	22 (39)	5 (6)	16 (28)	6 (7)	19 (33)	11 (13)	19 (33)
Very satisfied	28 (33)	23 (40)	28 (33)	28 (49)	29 (34)	32 (56)	27 (32)	30 (53)	24 (29)	26 (46)

Supplementary Table 12: Satisfaction of farmers (n = 86) and animal health workers (n = 57) with the laboratory services (diagnostic tests performed, affordability of laboratory tests, turnaround time for results delivery, confidence in test results, laboratory report quality, report confidentiality and value for money).

	**Diagnostic tests performed**		**Affordability of lab Tests**		**Turnaround time**		**Confidence in test results**		**Lab report quality**		**Report confidentiality**		**Value for money**	
	**Farmers**	**AHW**	**Farmers**	**AHW**	**Farmers**	**AHW**	**Farmers**	**AHW**	**Farmers**	**AHW**	**Farmers**	**AHW**	**Farmers**	**AHW**
	**n (%)**	**n (%)**	**n (%)**	**n (%)**	**n (%)**	**n (%)**	**n (%)**	**n (%)**	**n (%)**	**n (%)**	**n (%)**	**n (%)**	**n (%)**	**n (%)**
I'm not sure	25 (29)	2 (4)	23 (27)	2 (4)	23 (27)	2 (4)	22 (26)	2 (4)	22 (26)	3 (5)	25 (30)	5 (9)	22 (26)	3 (5)
Neither satisfied nor dissatisfied	22 (26)	4 (7)	22 (26)	7 (13)	23 (27)	7 (12)	23 (27)	4 (7)	23 (27)	4 (7)	26 (31)	7 (12)	23 (27)	6 (11)
Slightly dissatisfied		5 (9)	6 (7)	4 (7)	2 (4)	4 (7)		3 (5)		1 (2)		0	2 (2)	2 (4)
Very dissatisfied		1 (2)	3 (4)	1 (2)	1 (1)	2 (4)		1 (2)		0		0	1 (1)	1 (2)
Slightly satisfied	17 (20)	27 (47)	12 (14)	28 (50)	15 (18)	23 (40)	8 (10)	20 (36)	11 (13)	24 (42)	6 (7)	9 (16)	14 (17)	26 (46)
Very satisfied	21 (25)	18 (32)	19 (22)	14 (25)	21 (25)	19 (33)	31 (37)	26 (46)	28 (33)	25 (44)	27 (32)	36 (63)	22 (26)	19 (33)

Supplementary Table 13: Satisfaction of farmers (n = 86) and animal health workers (n = 57) with the post-test laboratory service (clarity of results, advice for vet referral, prescription intervention).

**Rate**	**Clarity of Results**		**Advice for Vet Referral**		**Prescription Intervention**	
	**Farmers**	**AHW**	**Farmers**	**AHW**	**Farmers**	**AHW**
	**n (%)**	**n (%)**	**n (%)**	**n (%)**	**n (%)**	**n (%)**
I'm not sure	24 (28)	2 (4)	21 (25)	4 (7)	23 (27)	3 (5)
Neither satisfied nor dissatisfied	21 (25)	5 (9)	23 (27)	5 (9)	24 (28)	8 (14)
Slightly dissatisfied	1 (1)	5 (9)	2 (2)	4 (7)	1 (1)	3 (5)
Slightly satisfied	11 (13)	22 (39)	13 (15)	17 (30)	12 (14)	21 (38)
Very dissatisfied	1 (1)	2 (4)	1 (1)	3 (5)		2 (4)
Very satisfied	27 (32)	21 (37)	25 (29)	23 (41)	25 (29)	19 (34)
