# Supplementary material for: A Survey of Priority Livestock Diseases and Laboratory Diagnostic Needs of Animal Health Professionals and Farmers in Uganda
Source: Front Vet Sci. 2021 Sep 23;8:721800. doi: 10.3389/fvets.2021.721800 (PMC8494769; doi:10.3389/fvets.2021.721800)
Supplement: Supplementary file 2 [file Data_Sheet_2.pdf]

Supplementary Data Sheet 2: Survey

Start of Block: Introduction questions

Please provide your consent to participate in this study and proceed.

○ Yes, I consent (1)

○ No, I do not consent (2)

Skip To: End of Survey If Dear Sir/Madam, We kindly invite you to participate in this online survey that seeks to assess... = No, I do not consent

Q2 Where is your **usual** place of **work**. Please select the **most applicable REGION (or place).**

▼ Acholi (10) ... Other (27)

Display This Question:

If Where is your usual place of work. Please select the most applicable REGION (or place). = Other

Q3 If you selected 'other' region, please specify the **region** below.

____

Q4 Where is your **usual** place of **work**. Please select the **most applicable DISTRICT.**

▼ Abim (11) ... Other (141)

Display This Question:

If Where is your usual place of work. Please select the most applicable DISTRICT. = Other

Q5 If you selected 'other' district, please specify the **district** below.

____

Q6 Where is your **usual** place of **RESIDENCE**. Please select the most applicable **REGION (or place).**

▼ Acholi (10) ... Other (27)

Display This Question:

If Where is your usual place of RESIDENCE. Please select the most applicable REGION (or place). = Other

Q7 If you selected 'other' region, please specify the **region** below.

____

Q8 Please indicate your age group:

- ○ 25 years or less (1)
- ○ 26-35 years (2)
- ○ 36-45 years (3)
- ○ 46-55 years (4)
- ○ 56 years or above (6)
- ○ Prefer not to say (5)

Q9 What is your gender?

- ◯ Male (1)
- ◯ Female (2)
- ◯ Prefer not to say (4)

Q10 Which of the following **best** describes your **profession at present**? Please select **one.**

- ◯ Veterinarian (1)
- ◯ Animal Production Officer or Animal Husbandry Officer (4)
- ◯ Farmer (2)
- ◯ Laboratory technologist or technician (7)

Display This Question:

If Which of the following best describes your profession at present? Please select one. = Laboratory technologist or technician

Q11 If you selected laboratory technologist or technician, which of the following **best** describes the **lab** in which your work at present? Please select one.

- ◯ Private lab (1)
- ◯ Academia (research/teaching) lab (6)
- ◯ Public/ non-government lab (4)
- ◯ Government lab (5)
- ◯ Other, please specify (9) ____

Display This Question:

If Which of the following best describes your profession at present? Please select one. = Veterinarian

Or Which of the following best describes your profession at present? Please select one. = Animal Production Officer or Animal Husbandry Officer

Q12 If you work as an animal health professional, which of the following **best** describes your employment sector at present?

- ○ Private practice (1)
- ○ Academia (research/teaching) (4)
- ○ Non-governmental organisation employee (2)
- ○ Government employee (3)
- ○ Not formally employed currently (5)
- ○ Not applicable (6)
- ○ Other, please specify (7) ____

End of Block: Introduction questions

Start of Block: Questions for lab workers ONLY

Q13 When was your laboratory established? (If you don't know please write 'not known' in the text boxes)

- ○ Year (1) ____
- ○ Month (2) ____

Q14 For how many years have **you worked** in the lab?

- 0-5 years (1)
- 6-10 years (2)
- 11-20 years (3)
- Over 20 years (5)
- Prefer not to say (4)

---

*

Q15 On **average**, approximately how many **total** samples do you receive **per week**?

____

Q16 On **average**, approximately how many samples from each of the following animal species are submitted per week?

Cattle (1)	▼ 0 (1) ... Over 100 (5)
Chickens (2)	▼ 0 (1) ... Over 100 (5)
Goats or sheep (3)	▼ 0 (1) ... Over 100 (5)
Pigs (4)	▼ 0 (1) ... Over 100 (5)
Horses, donkeys or mules (7)	▼ 0 (1) ... Over 100 (5)
Dogs (9)	▼ 0 (1) ... Over 100 (5)
Cats (10)	▼ 0 (1) ... Over 100 (5)
Turkeys, geese or ducks (11)	▼ 0 (1) ... Over 100 (5)
Fish/aquaculture (6)	▼ 0 (1) ... Over 100 (5)
Others (please specify species) (8)	▼ 0 (1) ... Over 100 (5)

Q17 Please select the **most common** tests requested by clients. Please select all that apply.

- ☐ Post-mortem examination (4)
- ☐ Microbiological culture (7)
- ☐ Microbiological culture and antimicrobial sensitivity testing (8)
- ☐ Serology (9)
- ☐ Complete Blood Count (10)
- ☐ Blood smear (11)
- ☐ Cytology/ histology examination (12)
- ☐ Polymerase Chain Reaction (PCR) (13)
- ☐ ⊗None of the above (14)
- ☐ ⊗Don't know (15)
- ☐ Other, please specify (16) ____

Q18 What are the **FIVE most common diseases** tested for in your laboratory? Please list **no more than FIVE**.

- 1 (1) ____
- 2 (2) ____
- 3 (3) ____
- 4 (4) ____
- 5 (5) ____

Q19 Which **diagnostic tests** are requested by your clients but you are **unable to provide**? Please list. (If not applicable please write 'none')

____

____

____

____

____

Q20 Which **diagnostic tests** would you like to have available at your lab to improve the range of services on offer? Please list the specific **test names**. (If not applicable please write 'none')

____

____

____

____

____

Q21 Which **diseases** would you like to be able to test for at your lab that you are currently **unable to test** for? Please list the specific **pathogen names**. (If not applicable please write 'none')

____

____

____

____

____

Q22 Please list the instruments/ equipment **most often used** in your lab?

____

____

____

____

____

Q23 To your knowledge, have these instruments/equipment been **calibrated** in the **last 2 years**?

- ○ Yes, all have been calibrated (5)
- ○ Some have been calibrated (1)
- ○ No (2)
- ○ I don't know (3)
- ○ Not applicable (4)

Q24 To your knowledge, have these instruments/equipment been **serviced** in the **last 2 years**?

- ○ Yes, all have been calibrated (1)
- ○ Some have been calibrated (5)
- ○ No (2)
- ○ I don't know (3)
- ○ Not applicable (4)

Display This Question:

If To your knowledge, have these instruments/equipment been serviced in the last 2 years? = Some have been calibrated

Or To your knowledge, have these instruments/equipment been serviced in the last 2 years? = No

Q25 Please briefly describe your reasons for the response given in the previous question.

____

____

____

____

____

Q26 Do you think clinicians (i.e. vets, animal health professionals etc.) appreciate the value of laboratory diagnostics?

- ○ Yes (1)
- ○ No (2)
- ○ I'm not sure (3)

Q27 Do you think **farmers** appreciate the value of laboratory diagnostics?

- Yes (1)
- No (2)
- I'm not sure (3)

Q28 Do you think **farmers** are willing to pay for diagnostic services?

- Yes (1)
- No (2)
- I'm not sure (3)

Q29 Do you think it is possible to run a veterinary diagnostic lab **profitably?**

- Yes (1)
- No (2)
- I'm not sure (3)

Display This Question:

If Do you think it is possible to run a veterinary diagnostic lab profitably? = Yes

Or Do you think it is possible to run a veterinary diagnostic lab profitably? = No

Q30 Please briefly describe your reasons for the response given to the previous question.

____

Q31 What are the **main challenges** you have faced that affect your ability to **use or offer** diagnostic services? Please briefly **list** the main challenges.

____

____

____

____

____

Q94 Have you ever carried out customer surveys/marketing to understand the diagnostic needs of your clients?

- ○ Yes (1)
- ○ No (2)
- ○ I'm not sure (3)

Q98 In your opinion, what do you consider the most important factors that determine whether clients will submit samples to a lab or not? Please rate the following factors by their importance to your decision.

	Not important (1)	Neither important or unimportant (neutral) (2)	Slightly important (3)	Very important (4)	Not sure (5)
Location and accessibility (proximity of lab to their farm/ home) (1)	○	○	○	○	○
Accreditation (National or international) of lab (4)	○	○	○	○	○
Organization and cleanliness of lab & building (5)	○	○	○	○	○
Reception area (someone present & space to sit) (6)	○	○	○	○	○
Availability of laboratory staff during working hours & beyond (7)	○	○	○	○	○
Professionalism, respect and appreciation of lab staff towards clients (8)	○	○	○	○	○
Guiding and communication between staff and clients on samples submitted (9)	○	○	○	○	○
Range of diagnostic tests available to clients (10)	○	○	○	○	○

Willingness to pay for lab tests and value for money (11)					
Turn-around time for tests done & results given (12)					
Confidence in test result (13)					
Quality of lab report (completeness, comprehension etc) and clear explanation of test results (14)					
Confidentiality of lab reports (15)					
Affordability of services (16)					
Prescription of intervention by clinician attached to the lab or referral to a vet clinician (17)					
Availability of sample collection, preservation & transportation materials (18)					

Q99 Of the factors you classified as 'very important' in the previous question, please select the **FIVE** you would **prioritise** as being the **most important**. Please select **no more than five** options.

In your opinion, what do you consider the most important factors that determine whether clients w... = Location and accessibility (proximity of lab to their farm/ home) [Very important]

☐ Location and accessibility (proximity of lab to their farm/ home) (1)

In your opinion, what do you consider the most important factors that determine whether clients w... = Accreditation (National or international) of lab [Very important]

☐ Accreditation (National or international) of lab (4)

In your opinion, what do you consider the most important factors that determine whether clients w... = Organization and cleanliness of lab & building [Very important]

☐ Organization and cleanliness of lab & building (5)

In your opinion, what do you consider the most important factors that determine whether clients w... = Reception area (someone present & space to sit) [Very important]

☐ Reception area (someone present & space to sit) (6)

In your opinion, what do you consider the most important factors that determine whether clients w... = Availability of laboratory staff during working hours & beyond [Very important]

☐ Availability of laboratory staff during working hours & beyond (7)

In your opinion, what do you consider the most important factors that determine whether clients w... = Professionalism, respect and appreciation of lab staff towards clients [Very important]

☐ Professionalism, respect and appreciation of lab staff towards clients (8)

In your opinion, what do you consider the most important factors that determine whether clients w... = Guiding and communication between staff and clients on samples submitted [Very important]

☐ Guiding and communication between staff and clients on samples submitted (9)

In your opinion, what do you consider the most important factors that determine whether clients w... = Range of diagnostic tests available to clients [Very important]

☐ Range of diagnostic tests available to clients (10)

In your opinion, what do you consider the most important factors that determine whether clients w... = Willingness to pay for lab tests and value for money [Very important]

☐ Willingness to pay for lab tests and value for money (11)

In your opinion, what do you consider the most important factors that determine whether clients w... = Turn-around time for tests done & results given [Very important]

☐ Turn-around time for tests done & results given (12)

In your opinion, what do you consider the most important factors that determine whether clients w... = Confidence in test result [Very important]

☐ Confidence in test result (13)

In your opinion, what do you consider the most important factors that determine whether clients w... = Quality of lab report (completeness, comprehension etc) and clear explanation of test results [Very important]

☐ Quality of lab report (completeness, comprehension etc) and clear explanation of test results (14)

In your opinion, what do you consider the most important factors that determine whether clients w... = Confidentiality of lab reports [Very important]

☐ Confidentiality of lab reports (15)

In your opinion, what do you consider the most important factors that determine whether clients w... = Affordability of services [Very important]

☐ Affordability of services (16)

In your opinion, what do you consider the most important factors that determine whether clients w... = Prescription of intervention by clinician attached to the lab or referral to a vet clinician [Very important]

☐ Prescription of intervention by clinician attached to the lab or referral to a vet clinician (17)

In your opinion, what do you consider the most important factors that determine whether clients w... = Availability of sample collection, preservation & transportation materials [Very important]

☐ Availability of sample collection, preservation & transportation materials (18)

Q32 Based on your experience of the veterinary diagnostic services available in your area, what are the **key aspects/problems** that **need to be improved** in order to attract more clients & improve client satisfaction.
Please briefly list the key aspects/problems.

____

Q33 Have you attended a continuous professional training in the **last 2 years**?

- ◯ Yes (1)
- ◯ No (2)

Display This Question:

If Have you attended a continuous professional training in the last 2 years? = Yes

Q34 If yes, please briefly detail the training you attended.

Display This Question:

If Have you attended a continuous professional training in the last 2 years? = No

Q35 If no, please briefly explain reasons behind this (e.g. not available/ not needed).

End of Block: Questions for lab workers ONLY

Start of Block: Questions for animal health professionals/farmers etc.

Q36 How many years of work/practice experience do you have? Please select **one**.

- 0-5 years (1)
- 6-10 years (2)
- 11-20 years (3)
- Over 20 years (5)
- Prefer not to say (4)

Display This Question:

If Which of the following best describes your profession at present? Please select one. = Veterinarian

Or Which of the following best describes your profession at present? Please select one. = Animal Production Officer or Animal Husbandry Officer

Q37 If applicable, which type of practice do you **predominantly** work in **at present**? Please select **one.**

- ◯ Small animal practice (dogs, cats) (1)
- ◯ Poultry practice (chickens, turkey etc.) (6)
- ◯ Ruminants (cattle, sheep, goats etc.) (3)
- ◯ Other livestock practice (swine, donkey, camel etc.) (10)
- ◯ Fish/aquaculture practice (7)
- ◯ Exotic practice (rabbits, parrots, tortoise, snakes etc.) (8)
- ◯ Wildlife practice (9)
- ◯ Mixed practice (two or more of the practices listed above) (2)
- ◯ Other, please specify (4) ____
- ◯ I do not work in practice (5)

Display This Question:

If Which of the following best describes your profession at present? Please select one. = Farmer

Q38 If you work in livestock production, which of the following **best** describes the **production sector** in which you work? Please select **one.**

- ◯ Cattle farming (1)
- ◯ Pig farming (2)
- ◯ Poultry farming (3)
- ◯ Other, please specify (10) ____

Display This Question:

If Which of the following best describes your profession at present? Please select one. = Farmer

Q39 If you work in livestock production **which animals** do you keep on your farm **at present**? Please select all that apply.

- ☐ Cattle (1)
- ☐ Chickens (2)
- ☐ Goats (3)
- ☐ Pigs (4)
- ☐ Sheep (5)
- ☐ Horses, donkeys or mules (6)
- ☐ Ducks or geese (7)
- ☐ Turkeys (8)
- ☐ Fish/aquaculture (11)
- ☐ Other, please specify (10) ____

Display This Question:

If Which of the following best describes your profession at present? Please select one. = Farmer

Q40 Please specify the **approximate number** of each animal species you keep on your farm **at** present.

If you work in livestock production which animals do you keep on your farm at present? Please sel... = Cattle

○ Cattle (1) ____

If you work in livestock production which animals do you keep on your farm at present? Please sel... = Chickens

○ Chickens (4) ____

If you work in livestock production which animals do you keep on your farm at present? Please sel... = Goats

○ Goats (5) ____

If you work in livestock production which animals do you keep on your farm at present? Please sel... = Pigs

○ Pigs (6) ____

If you work in livestock production which animals do you keep on your farm at present? Please sel... = Sheep

○ Sheep (7) ____

If you work in livestock production which animals do you keep on your farm at present? Please sel... = Horses, donkeys or mules

○ Horses, donkeys or mules (8)

____

If you work in livestock production which animals do you keep on your farm at present? Please sel... = Ducks or geese

○ Ducks or geese (9) ____

If you work in livestock production which animals do you keep on your farm at present? Please sel... = Turkeys

○ Turkeys (10) ____

If you work in livestock production which animals do you keep on your farm at present? Please sel... = Fish/aquaculture

○ Fish/aquaculture (13) ____

If you work in livestock production which animals do you keep on your farm at present? Please sel... = Other, please specify

◯ Other (12) ____

Display This Question:

If Which of the following best describes your profession at present? Please select one. = Farmer

Q41 If you work in livestock production, do you belong to any of the following farmer groups? Please select all that apply.

▢ Livestock Development Forum (LDF) (1)

▢ Goat farmers association (4)

▢ Poultry farmers association (5)

▢ Pig farmers association (6)

▢ Other, please specify (7) ____

▢ ⊗None (9)

▢ ⊗Not applicable (8)

Q42 How many times in the **last six months** did you submit samples to the laboratory to aid your diagnosis or diagnosis of illness observed?

- ○ Never (1)
- ○ 1-2 times in six months (3)
- ○ 3-6 times in six months (4)
- ○ 1-3 times per month (6)
- ○ Over 3 times a month (8)
- ○ Don't know (10)

Display This Question:

If How many times in the last six months did you submit samples to the laboratory to aid your diagno... != Never

Q43 From which species of animals do you most frequently submit samples to laboratories? Select a **maximum of three.**

- ☐ Cattle (1)
- ☐ Chickens (2)
- ☐ Goats (3)
- ☐ Pigs (4)
- ☐ Sheep (5)
- ☐ Horses, donkeys or mules (6)
- ☐ Ducks or geese (7)
- ☐ Turkeys (9)
- ☐ Other, please specify (8) ____

Display This Question:

If Which of the following best describes your profession at present? Please select one. = Veterinarian

Or Which of the following best describes your profession at present? Please select one. = Animal Production Officer or Animal Husbandry Officer

Q44 What types of tests did you **commonly request** when submitting samples to labs **for clinical diagnosis**, in the **last six months**? Check all that apply.

- ☐ Post-mortem examination (1)
- ☐ Microbiological culture (2)
- ☐ Microbiological culture and antimicrobial sensitivity testing (3)
- ☐ Serology (4)
- ☐ Complete Blood Count (7)
- ☐ Blood smear (11)
- ☐ Cytology/ histology examination (8)
- ☐ Polymerase Chain Reaction (PCR) (10)
- ☐ ⊗ Did not submit samples (5)
- ☐ ⊗ Don't know (9)
- ☐ Other, please specify (6) ____

Q45 The following questions will ask you to rate your level of satisfaction regarding your current veterinary diagnostic services (i.e. the lab facilities you most frequently submit samples to). Please answer honestly and openly to the best of your ability.

Q46

In regards to the laboratory facilities that you most frequently submit samples to, please rate your satisfaction of the following:

	Very satisfied (1)	Slightly satisfied (2)	Neither satisfied nor dissatisfied (3)	Slightly dissatisfied (4)	Very dissatisfied (5)	I'm not sure (6)
Laboratory location (i.e. conveniently located, accessible) (1)	○	○	○	○	○	○
Organization of the lab (is it organized) (3)	○	○	○	○	○	○
Cleanliness of the lab (4)	○	○	○	○	○	○
Reception area (e.g. someone present & space to sit) (5)	○	○	○	○	○	○

Q47 In regards to the **laboratory staff** at these facilities**,** please rate your satisfaction of the following:

	Very satisfied (1)	Slightly satisfied (2)	Neither satisfied nor dissatisfied (3)	Slightly dissatisfied (4)	Very dissatisfied (5)	I'm not sure (6)
Availability within working hours (1)	o	o	o	o	o	o
Staff appearance (e.g. professionally dressed) (2)	o	o	o	o	o	o
Staff demenour i.e. communication & welcoming of clients (3)	o	o	o	o	o	o
Respect & appreciation of clients (4)	o	o	o	o	o	o
Guiding clients on sample submission process & turnaround time (5)	o	o	o	o	o	o

Q48 In regards to the **tests carried out** by the labs, please rate your satisfaction of the following:

	Very satisfied (1)	Slightly satisfied (2)	Neither satisfied nor dissatisfied (3)	Slightly dissatisfied (4)	Very dissatisfied (5)	I'm not sure (6)
Range of diagnostic tests performed (1)	○	○	○	○	○	○
Affordability of lab tests (2)	○	○	○	○	○	○
Turn-around time for performing tests & result delivery (3)	○	○	○	○	○	○
Confidence in test results (4)	○	○	○	○	○	○
Quality of lab report (completeness/detail, comprehension etc) (5)	○	○	○	○	○	○
Confidentiality of lab reports (results kept confidential) (6)	○	○	○	○	○	○
Value for money (7)	○	○	○	○	○	○

Q49 In regards to the **quality of post-test services,** please rate your satisfaction of the following:

	Very satisfied (1)	Slightly satisfied (2)	Neither satisfied nor dissatisfied (3)	Slightly dissatisfied (4)	Very dissatisfied (5)	I'm not sure (6)
Explanation of the test results (i.e. clarity & detail provided) (1)	○	○	○	○	○	○
Advice on need for vet referral (2)	○	○	○	○	○	○
Prescription of treatments & intervention provided by the lab/ lab clinician (3)	○	○	○	○	○	○

End of Block: Questions for animal health professionals/farmers etc.

Start of Block: Questions for animal health professionals/farmers etc. continued

Q50 What do you consider the **most important factors** that determine whether you will submit samples to a lab or not? Please rate the following factors by their importance to your decision.

	Not important (1)	Neither important or unimportant (neutral) (2)	Slightly important (3)	Very important (4)	Not sure (5)
Location and accessibility (proximity of lab to my farm/ home) (1)	○	○	○	○	○
Accreditation (National or international) of lab (3)	○	○	○	○	○
Organization and cleanliness of lab & building (4)	○	○	○	○	○
Reception area (someone present & space to sit) (6)	○	○	○	○	○
Availability of laboratory staff during working hours & beyond (7)	○	○	○	○	○
Professionalism, respect and appreciation of lab staff towards clients (8)	○	○	○	○	○
Guiding and communication between staff and clients on samples submitted (12)	○	○	○	○	○
Range of diagnostic tests available to clients (13)	○	○	○	○	○

Willingness to pay for lab tests and value for money (14)	○	○	○	○	○
Turn-around time for tests done & results given (16)	○	○	○	○	○
Confidence in test result (17)	○	○	○	○	○
Quality of lab report (completeness, comprehension etc) and clear explanation of test results (18)	○	○	○	○	○
Confidentiality of lab reports (19)	○	○	○	○	○
Affordability of services (20)	○	○	○	○	○
Prescription of intervention by clinician attached to the lab or referral to a vet clinician (24)	○	○	○	○	○
Availability of sample collection, preservation & transportation materials (25)	○	○	○	○	○

Q51 Of the factors you classified as '<u>very important</u>' in the previous question, please select the **<u>top FIVE</u>** factors that you would **<u>prioritise</u>** as the **<u>most important.</u>**

What do you consider the most important factors that determine whether you will submit samples to... = Location and accessibility (proximity of lab to my farm/ home) [Very important]

☐ Location and accessibility (proximity of lab to my farm/ home) (1)

What do you consider the most important factors that determine whether you will submit samples to... = Accreditation (National or international) of lab [Very important]

☐ Accreditation (National or international) of lab (3)

What do you consider the most important factors that determine whether you will submit samples to... = Organization and cleanliness of lab & building [Very important]

☐ Organization and cleanliness of lab & building (4)

What do you consider the most important factors that determine whether you will submit samples to... = Reception area (someone present & space to sit) [Very important]

☐ Reception area (someone present & space to sit) (6)

What do you consider the most important factors that determine whether you will submit samples to... = Availability of laboratory staff during working hours & beyond [Very important]

☐ Availability of laboratory staff during working hours & beyond (7)

What do you consider the most important factors that determine whether you will submit samples to... = Professionalism, respect and appreciation of lab staff towards clients [Very important]

☐ Professionalism, respect and appreciation of lab staff towards clients (8)

What do you consider the most important factors that determine whether you will submit samples to... = Guiding and communication between staff and clients on samples submitted [Very important]

☐ Guiding and communication between staff and clients on samples submitted (12)

What do you consider the most important factors that determine whether you will submit samples to... = Range of diagnostic tests available to clients [Very important]

☐ Range of diagnostic tests available to clients (13)

What do you consider the most important factors that determine whether you will submit samples to... = Turn-around time for tests done & results given [Very important]

☐ Willingness to pay for lab tests and value for money (14)

What do you consider the most important factors that determine whether you will submit samples to... = Turn-around time for tests done & results given [Very important]

☐ Turn-around time for tests done & results given (16)

What do you consider the most important factors that determine whether you will submit samples to... = Confidence in test result [Very important]

☐ Confidence in test result (17)

What do you consider the most important factors that determine whether you will submit samples to... = Quality of lab report (completeness, comprehension etc) and clear explanation of test results [Very important]

☐ Quality of lab report (completeness, comprehension etc) and clear explanation of test results (18)

What do you consider the most important factors that determine whether you will submit samples to... = Confidentiality of lab reports [Very important]

☐ Confidentiality of lab reports (19)

What do you consider the most important factors that determine whether you will submit samples to... = Affordability of services [Very important]

☐ Affordability of services (20)

What do you consider the most important factors that determine whether you will submit samples to... = Prescription of intervention by clinician attached to the lab or referral to a vet clinician [Very important]

☐ Prescription of intervention by clinician attached to the lab or referral to a vet clinician (24)

What do you consider the most important factors that determine whether you will submit samples to... = Availability of sample collection, preservation & transportation materials [Very important]

☐ Availability of sample collection, preservation & transportation materials (25)

End of Block: Questions for animal health professionals/farmers etc. continued
