# Supplementary material for: A Survey of Priority Livestock Diseases and Laboratory Diagnostic Needs of Animal Health Professionals and Farmers in Uganda
Source: Front Vet Sci. 2021 Sep 23;8:721800. doi: 10.3389/fvets.2021.721800 (PMC8494769; doi:10.3389/fvets.2021.721800)
Supplement: Supplementary file 3 [file Data_Sheet_3.pdf]

Supplementary Data Sheet 3: Key informant guide

<u>Interview guide questions</u>

1. For how long have you worked in your organization?
   Probe: What is your role in veterinary diagnostic services in Uganda?

2. Who are the major clients of diagnostic services in Uganda?
   Probe: which diagnostic services do they demand?
   Probe: Give your view on what influences their willingness to demand and pay for the diagnostic services mentioned above?

3. What is your perception about the current situation of veterinary diagnostic services in Uganda?
   Probe: in your opinion, do you think the country's laboratory services are adequate, well-equipped with both facilities & competent human resources, quality of service provided Vz acceptable standards (by OIE & accrediting agencies)?

4. In your opinion, why are the existing government veterinary diagnostic laboratories struggling to stay sustainable?
   Probe: Are the government labs supported with necessary equipment, reagents/ consumables, human resource and administrative support to deliver services to clients/ farmers?

5. In your view, do you think that the number of private veterinary labs are adequate for the work available, please elaborate.
   Probe: how do you think the vet labs compare with the human health labs? Nos, performance.
   Probe: In your opinion, why are private veterinary diagnostic laboratories very few compared to human health?
   Probe: What should existing veterinary diagnostic laboratories do to attract more clients to utilize their services?

6. In your opinion, what are the most relevant diagnostic services demanded by clients in Uganda? Please explain your response.

7. How can farmers be encouraged to utilize veterinary diagnostic services in Uganda?

8. How can veterinary extension service providers be encouraged to utilize veterinary diagnostic services in Uganda?

9. Do you have any suggestions that you think will improve our work? Please kind provide them.

10. Do you have any questions for me? Please go ahead and ask.
