# Supplementary material for: A Survey of Priority Livestock Diseases and Laboratory Diagnostic Needs of Animal Health Professionals and Farmers in Uganda
Source: Front Vet Sci. 2021 Sep 23;8:721800. doi: 10.3389/fvets.2021.721800 (PMC8494769; doi:10.3389/fvets.2021.721800)
Supplement: Supplementary file 4 [file Data_Sheet_4.pdf]

Supplementary Data Sheet 4: Demographic details of key informants

Details of key informants interviewed in this study

KII:CODE	Interview date	Profession	Job description	Location
KII:01	07/07/2020	Veterinary Surgeon and Head of District Veterinary Laboratory	• Managing district veterinary diagnostic laboratory<br>• Animal disease diagnosis<br>• Treatment and vaccination<br>• Training farmers and advisory services<br>• Reporting diseases to district veterinary officer	Wakiso district
KII:02	09/7/2020	Farmer	• Rearing livestock<br>• Utilizes veterinary clinical and diagnostic services<br>• Participates in farmer training seminars	Mukono district
KII:03	14/7/2020	Veterinary Surgeon, Experienced Clinician, Frequent user of veterinary laboratory diagnostic services	• Animal disease diagnosis<br>• Treatment and vaccination<br>• Training of farmers and advisory services<br>• Reporting diseases to district veterinary officer	Sembabule district
KII:04	17/7/2020	Veterinary Surgeon and Head of Regional Veterinary Laboratory	• District production officer<br>• Supervise all veterinary staff at the district<br>• Animal disease diagnosis<br>• Treatment and vaccination<br>• Supervision of Regional Veterinary diagnostic laboratory<br>• Monitoring	Mbarara
KII:05	23/7/2020	Veterinary Surgeon and District Veterinary officer	• District veterinary officer<br>• Plans, implements, and monitors animal health and production activities at the district<br>• Supervise all veterinary staff at the district<br>• Issuance of animal movement permit<br>• Animal disease diagnosis<br>• Treatment and vaccination<br>• Report animal diseases to commissioner animal health	Nakaseke
KII:06	01/8/2020	Veterinary Surgeon and Assistant Commissioner for Inspections and Regulations	• Develop policies<br>• Inspection and certification of animals, animal products, abattoirs, and processing plants	Directorate of Animal Resources, Ministry of Agriculture, Animal Industry and Fisheries (MAAIF)

KII:07	2/9/2020	Veterinary Surgeon and Head of National Animal Disease Diagnostics and Epidemiology Center (NADEC)	• Laboratory management<br>• Animal disease outbreak investigation<br>• Reporting diagnostics to Commissioner Animal Health<br>• Training staff on SOPs for inspections	Directorate of Animal Resources, Ministry of Agriculture, Animal Industry and Fisheries (MAAIF)
KII:08	08/9/2020	Veterinary Surgeon, Senior Lecturer and Experienced Clinician	• Offering Clinical veterinary services to farmers<br>• Artificial insemination<br>• Training students<br>• Animal disease diagnostics	Makerere University College of Veterinary Medicine, Animal Resources and Biosecurity COVAB)
