# Supplementary material for: A Survey of Priority Livestock Diseases and Laboratory Diagnostic Needs of Animal Health Professionals and Farmers in Uganda
Source: Front Vet Sci. 2021 Sep 23;8:721800. doi: 10.3389/fvets.2021.721800 (PMC8494769; doi:10.3389/fvets.2021.721800)
Supplement: Supplementary file 5 [file Data_Sheet_5.pdf]

Supplementary Data Sheet 5: Key informants quotes

Extracted quotations for Key Informat Interviews - KII:01 - KII:08

	Themes	Codes
1	Livestock/poultry diseases and clientele	1. Livestock/poultry diseases and animal losses<br>2. Clientele
2	Challenges towards existing animal diagnostic services	1. Access to labs<br>2. Self-treatment on farms<br>3. Lab human resources
2	Additional code added to Theme 2	4. Laboratory equipment and supplies
3	Education and government policies	1. Education and training<br>2. Funding and Investment<br>3. By-laws and policies

Kll:01
Extracted quotations

Theme 1) Livestock - poultry diseases and clientele

Codes
1. Livestock - poultry diseases and animal losses
Kll:01- "Farmers usually come for lumpy skin disease and different types of diagnosis for FMD e.g. foot rot."
Kll:01- "Blood parasites especially tick-borne parasites, viral diseases and mastitis."
Kll:01- "Gain; farmers especially those doing commercial farming are very keen on incurring losses for example poultry farmers keeping layers are very keen on keeping the birds healthy and cattle keepers are keen on mastitis."

2. Clientele
Kll:01- "Poultry and dairy farmers."

Theme 2) Challenges towards existing animal diagnostic services

Codes
1. Access to labs
Kll:01- "I do not think that private veterinary labs are adequate because around here there are none, apart from those (i do not know if we should call them labs), that do mortem and a little but of microscopy, I think vets have not seen the motivation to put them up."
Kll:01- "Vets have to collect a sample; maybe cover another 30 miles the location makes it more difficult."
Kll:01- "Victims are scattered, there is no central point where we can bring clients together for diagnosis, or where you can put the lab and serve very many of them. I think it is the method of how we deliver services. For example, if I had a small animal clinic where a dog or a cat is brought, that is ok."

2. Self-treatment on farms
Kll:01- “Even if you say let’s carry out the diagnosis and confirm, the vet still wants to treat, instead of saying let me delay treatment for like an hour.”

3. Lab human resources
Kll:01- “The staff structure of the labs is still lacking, labs have lab technicians instead of lab technologists, who are mire competent.”
Kll:01- “In most cases you find that the CAO (Chief administrative officer) is not a scientist, so funding becomes a challenge.”

Additional code

4. Laboratory equipment and supplies
Kll:01- “The district labs are poorly equipped compared to the national or regional labs.”
Kll:01- “Lab supplies, equipment, reagents is around below 30%.”

<u>Theme 3) Education and Government policies</u>

Codes
1. Education and training
Kll:01- “People in charge do not understand or appreciate the relevance of what we do, he does not understand the concept behind what we do. People at that level of governance do not appreciate the relevance of confirmatory diagnosis, it is not taken as a priority.”
Kll:01- “Demonstrating how useful the diagnostic approach is, showing its benefits. For example, a cow with subclinical mastitis giving 12litres after treatment, the cow gives another 4 litres, such a farmer will never stop.”
Kll:01- “Sensitisation right from the vets, extension workers and then farmers. Farmers are willing to pay for services, so it’s a matter of sensitising people.”

2. Funding and Investment
Kll:01- “Community service providers from the ministry and other organisations like JICA no longer come, which contributes to the decline in this last one year for veterinary DX services.”
Kll:01- “Someone complained that the lab was using up a lot of electricity, so they switched it off and we could not maintain the cold chain because of lack of power, sometimes the problem remains over weekend and long periods of time.”
Kll:01- “We have been suggesting that if we cannot be served by UMEME, let us buy a solar panel, but now for 4 years down the road, the system is not yet up.”

3. By-laws and policies

N/A

<u>Kll:02</u>
<u>Extracted quotations</u>

<u>Theme 1) Livestock - poultry diseases and clientele</u>

Codes
1. Livestock - poultry diseases and animal losses
Kll:02- “poultry diseases”.

2. Clientele
Kll:02- “I know only vets.”

<u>Theme 2) Challenges towards existing animal diagnostic services</u>

Codes
1. Access to labs
Kll:02- “We have only one lab in Mukono, but it needs a farmer to travel quite a long distance to access services.”
Kll:02- “I have never heard of any private lab apart from Makerere. So i guess there is demand for services to the lower community.”
Kll:02- “Our biggest farm is in highlands in Koome in Mukono, I don’t know how the lab technician can reach there because it’s tough getting there.”
Kll:02- “In Mukono in each and every trading centre there is a human lab, but we have only one vet lab at the district.”
Kll:02- “Diagnostic services need mobile clinics where by it is easier to go with the lab equipment at the farm and make the diagnosis. When you collect samples to the farm sometimes transportation of samples to the lab is not good.”
Kll:02- “To establish more facilities such that they are easily accessed within their reach.”

2. Self-treatment on farms
Kll:02- “It is cheaper to pay for the service than using try and error, they just come probably thinking this is ECF, they give the drug which is expensive then later the animal does not improve they propose something else. So, it’s cheaper on a long run to pay for diagnostic services other than using the try and error approach.”
Kll:02- “8/10 of service providers will never advise a farmer on lab use before treatment. They use signs and symptoms to treat.”

3. Lab human resources
Kll:02- “There could be few professionals in vet diagnostics which could explain why there are very few private veterinary diagnostic laboratories.”
Kll:02- “Inadequate staff here in Mukono where we have one staff if he is on leave, sick or join another organisation, the service won’t be sustained.”
Kll:02- “First human resources are very little, even lab almost here is operated by one person which make it insufficient to serve the entire community of Mukono.”
Kll:02- “If vet professionals can train some youths to act as voluntary lab technicians in terms of collecting samples and delivering them quickly to the lab , so as to ease pressure on the few staff, because farmers find it difficult to travel to the lab, deliver sample, wait and travel back. If there is a middle person pay them little money to do this.”

Additional code

4. Laboratory equipment and supplies
Kll:02- “Sometimes equipment and materials are not enough for instance you find reagents to diagnose some diseases are not available and we are referred to Makerere.”

Theme 3) Education and Government policies

Codes

1. Education and training

Kll:02- “ Private veterinary diagnostic laboratories are very few probably because the service is still new, and people are sceptical to invest in it, due to lack of knowledge or awareness.”
Kll:02- “There should be government intervention in training of personnel and equipping of labs especially by ministry of agriculture.”
Kll:02- “Institutions like Makerere, colleges like Bukalasa etc should establish these diagnostic services at their colleges. This will increase the number of labs available and expose their students.”
Kll:02- “It should be embedded in the training in colleges and universities on the advantages of lab diagnosis before treatment.”
Kll:02- “They should have veterinary lab exhibition week where they could go in an area and provide services whether free, so that people can become aware.”
Kll:02- “There should be a collaboration between labs and drug manufacturers such that on the drug bottle if they could write that before you use this drug. Make sure your animal is tested so that the farmers can be able to demand.”
Kll:02- “It is because of lack of awareness and sensitisation of the farming community.”
Kll:02- “There should be mass sensitisation using mass media because most farmers are not aware of the service.”

2. Funding and investment

Kll:02- “The government budget allocated to agriculture is too small. This year agriculture received about 3.3% which is very little.”
Kll:02- “Farmers have an attitude regarding government services which are given free of charge, hence demanding user fee from a farmer would be a problem and anything given for free cannot be sustainable.”
Kll:02- “The government needs to subsidise services to make them affordable to all farmers.”
Kll:02- “The costs of establishing more vet labs could be hampering.”
Kll:02- “People engaged in the business should see this opportunity and be able to invest in lab services.”

3. By-laws and policies

Kll:02- “Decision making politicians may not see the importance in stimulating and supporting animal production.”
Kll:02- “There should be collaboration in extension service sectors e.g. agriculture, fisheries, entomology, should also be aware and promote animal testing whenever they go to the farm and find that you have animals.”

Kll:03
Extracted quotations

Theme 1) Livestock - poultry diseases and clientele

Codes

1. Livestock - poultry diseases and animal losses

Kll:03- “worm and TB (Para Tuberculosis), FMD, Papilloma which has become a big problem, because we do not know whether we still have the real papilloma virus because there is some which is very aggressive, it can attack the whole herd.”
Kll:03- “Trypanasomes and TBDS, hermoparasites, abortions and fetal deaths.”
Kll:03- “Economic losses due to abortions and fertility challenges disturbs the farmers a lot, they have to seek for services because they have no solution when the animals do not conceive.”

2. Clientele
Kll:03- “Farmers.”

<u>Theme 2) Challenges towards existing animal diagnostic services</u>

Codes
1. Access to labs
Kll:03- “There are some test that are supposed to be done on farm like trichomonas, because of the time limit that is given to collect the sample and then examine it (the 3 hours), you cannot take it to the lab when it is very far, so we need some field lab services which can examine there and then.
Kll:03- “My sub county Lugusulu, six extension staff and not even a motorcycle, no transport means, how can we reach farmers?”
Kll:03- “Accessibility to farmers is important, farmers call for help and someone never comes so they get disappointed and sell off the animals.”
Kll:03- “Facilitation in terms of transport, vet services transport is very poor.”

2. Self-treatment on farms
Kll:03- “The cost of treatment is becoming high without knowing what they are treating, because at first they gamble with the drugs and drugs are expensive like ECF, so when the cost becomes high they opt for diagnosis.”
Kll:03- “Vets should not rush to treat, vets should discourage the habit of rushing treatment without lab findings.
Kll:03- “Self-treatment is a practice which I found here, most farmers are practitioners, some farmers would even tell you like deworming, ‘that one you give it 80ml,’ they would even direct you on how much to give, they will add a dose according to the prognosis of the diseases.”
Kll:03- “When the animal is very poorly they will say give it more drug, but the animal is very weak, but the farmer says add it more drug since it is very sick. They do not know that it does not depend on how sick the animal is, they do not know it is about the live weight of the animal.”
Kll:03- “when a farmer abuses self-treatment practices he faces losses, the animals die and next time he will call for a professional after feeling the shock of seeing his animals die.”

3. Lab human resources
Kll:03- “Ministry policy, failure to equip staff, staff are not equipped hence cannot access farmers.”
Kll:03- “In the Nabitanga lab there is only one technician, you will find him overwhelmed when he goes for a workshop, no one is available to attend to the farmers.”

Additional code

4. Laboratory equipment and supplies
Kll:03- “We are still lacking on technology because there are some diseases we cannot do much with for example, viral diseases like FMD, pustular vulvovaginitis and it is very common, but we usually just guess.”
Kll:03- “Sometimes reagents aren’t there like for CBPP, even sometimes we have fake reagents on market.”
Kll:03- “Laboratory services are not well equipped, we just have buildings but no equipment.”
Kll:03- “We are lacking is the equipment and technology, the problem is consistency on and off challenges you find this time they can do Leptospirosis and next time they do not have reagents, we have an under supply of reagents.”
Kll:03- “Farmers are moving away from that historical traditional veterinary practice, they are now becoming ‘.com’ they are moving with the technology.”
Kll:03- “Laboratory supplies are there in Ssembabule, you have to go to Mbarara or Kampala to get them supplies like a vactutainer, formalin, post mortem tissue collection materials, cool box, ice or the cold chain itself is not available.”
Kll:03- “Technologies that conform to their modes of practice (farmer), like now I was one time consulting about albendazole abuse, so sometimes technical people make research and find out a solution, but does it customise well with the modes of practice of the farmers?”
Kll:03- “Provide basic equipment like vacutainers, postmortem dishes, preservation reagents, etc.”

<u>Theme 3) Education and Government policies</u>

Codes
1. Education and training
Kll:03- “Preservation and staff, the vets cannot collect and preserve samples. The do not know what to collect, for what purposes, procedures for example someone to collect EDTA for Brucellosis. They lack hands on training.”
Kll:03- “Staffs need refresher courses to collect rightful samples, because if you take a fake sample, you get fake results.”
Kll:03- “Staff from NARO, MAK, and MAAIF are very good, they have got some good training, people who are fresh from institutions are still lacking and need some mentorship.”
Kll:03- “Training both field and lab staff to be competent to do their job.”
Kll:03- “Farmers learn by results, if there are two sets of farmer, one is using laboratory services and the other is not using, these farmers get informal education. They learn from one another.”
Kll:03- “The more farmers are trained the more they visualise lab services as being very important.”
Kll:03- “Capacity building and giving all facilities necessary (training them on sample collection and basic lab like making impression smears and microscopy), this can be stimulating them into lab services.”
Kll:03- “Laboratory should be built in form of research. They should not stop at examining only what has been brought to the lab, but they should continue to do further research. You may find something of importance when actually you are looking for something else, which actually attract research.”

Kll:03- “Labs should have field days like promotions and demonstrate to the farmers see these things how bacteria, or typos or clostridia or theileria look like. Promotion and exhibition increasing awareness to diseases and increase exposure of the farmers to the reality.
Kll:03- “Sensitise farmers on the role of diagnostics and specific treatment to avoid associated losses.”

2. Funding and investment
Kll:03- “Farmers are not about cost; the farmers issue is solving the problem that he has encountered on his farm. I have seen farmers willing to pay up to 1.5 million shilling for lab services.”
Kll:03- “Laboratory services are there to help farmers to reduce costs in terms of animal losses and in terms of expenditure, by reducing cost of production and losses due to diseases.”
Kll:03- “Sometimes when power goes off, fridges stop. In Sembabule am now making my ice and give them field vets free of charge.”

3. By-laws and policies

N/A

Kll:04
Extracted quotations

Theme 1) Livestock - poultry diseases and clientele

Codes
1. Livestock - poultry diseases and animal losses
Kll:04- “Brucellosis, sometimes FMD.”
Kll:04- “The major reason they come is because of the losses they incur.”

2. Clientele
Kll:04- “The major clients are vets, those who want to find out what is in the animals before they treat. It is their own initiative to refer to the labs so that they do proper treatments.”
Kll:04- “Farmers, the breeders, first time buyers also rely on the labs to know the status of the animals before they are sold or brought.”
Kll:04- “Investors, researchers who use the services for storage, or help out in the field. We recently had some clients from outside Uganda; Rwanda, Burundi, who were screening animals before export.”

Theme 2) Challenges towards existing animal diagnostic services

Codes
1. Access to labs
Kll:04- “Locations of some labs and lack of linkages like the lab in Entebbe which depends on samples from villages. There are no strong linkages, all experts are in Entebbe and none in villages.”

Kll:04- “Services are not run where they are very much required, i.e. in the villages where farmers are located.”
Kll-04- “As a country that depends so much on livestock, I feel that we still need more services.”

2. Self-treatment on farms
Kll:04- “They have realised that treatment without proper diagnosis is expensive in terms of the cost of treatment and losses incurred due to guess work.”
Kll:04- “People were allowed to dot seize the syringe and start doing self-treatments on their farms, affecting accuracy in diagnosis.”
Kll:04- “Move away from traditional methods of farming, they don’t know that treating without diagnosis is dangerous.”

3. Lab human resources
Kll:04- “People who supervise labs do not have enough capacity to do so.”

Additional code

4. Laboratory equipment and supplies
Kll:04- “We lack regents here, there are some kits or reagents which are very expensive and you find it very difficult because we can only examine a few animals, with the kits we have.”
Kll:04- “We cannot purchase a kit of brucella, FMD.”
Kll:04- “When it comes to capacity in terms of equipment and personnel, I think we still need more.”

<u>Theme 3) Education and Government policies</u>

Codes
1. Education and training
Kll:04- “We need educate those who are doing self-treatment.”
Kll:04- “People have been surviving with animals without a diagnosis - vaccines for a very long time. The animals don’t necessarily improve, and the farmers don’t know that improvement is necessary as time goes by.”
Kll:04- “Political awareness and goodwill, decision makers do not realise the importance of diagnostic services, education is key.”
Kll:04- “Veterinary extension service providers need to know sample handling and collection, also need to have knowledge of the test required.”
Kll:04- “When people are being trained, emphasis should be on areas which should be emphasises for example, if you know diagnosis takes 50% of treatment it should be emphasised during class education.”
Kll:04- “Competitions, prizes, rewards for farmers/clients supporting the operations of diagnostic testing and lab services, popularising their farms and looking for success stories.”
Kll:04- “The element of one health has not been promoted very well.”
Kll:04- “Sensitisation and awareness to decision makers about disease development.”
Kll:04- “Political awareness and goodwill, decision makers are not sensitised.”

2. Funding and investment
Kll:04- “There is lack of a vibrant private sector, If we had practicing vets who were paying taxes and well as registered.”

Kll:04- “There is a farmer who cannot afford he services to sustain this private vet, and the private vet does not have the capacity to package himself for farmer.”

3. By-laws and policies
Kll:04- “We need by-laws such as, animals should not be moved without testing, followed by implementation.”
Kll:04- “Remove unnecessary taxes on kits.”
Kll:04- “We should record our animals to know what we have in the country such as a national record.”
Kll:04- “A board of farmers either as registered members recognised for supporting lab and use of diagnostic services.”

Kll:05
Extracted quotations

Theme 1) Livestock - poultry diseases and clientele

Codes
1. Livestock - poultry diseases and animal losses
Kll:05- “We have diseases like paralysis, eye problems and anaplasmosis, especially with those fake drugs on the market.”
Kll:05- “Tick borne diseases and pregnancy related diseases are very common.”

2. Clientele
Kll:05- “co-operative societies, but mainly people with big farms, especially dairy farms since we are now dealing with improved breeds, we now require the services.”

Theme 2) Challenges towards existing animal diagnostic services

Codes
1. Access to labs
Kll:05- “Labs are very far away.”
Kll:05- “We need distribution of labs in several locations in the sub countries.”
Kll:05- “Bring the services to where the farmers stay and so we can sensitise them.”
Kll:05- “Bring the services where the people need them, where the demand is.”

2. Self-treatment on farms
Kll:05- “Farmers attitude they usually go with preventative treatment. They do not wait for animals to get sick.”
Kll:05- “Farmers spend more when they do not used diagnostic services, because the vets use drugs for all diseases due to changes in the symptoms and in the end it is very expensive compared to when you first check and treat what you know.”

3. Lab human resources
Kll:05- “It is only recently that a lab in Butalama has been established and it is understaffed so it is ineffective.”

Additional code

4. Laboratory equipment and supplies
Kll:05- “In case of out breaks like FMD we need equipment like vacutainers, needles to collect blood.”
Kll:05- “We need more facilities and equipment to go to the field.”

Theme 3) Education and government policies

Codes
1. Education and training
Kll:05- “Training should be available to all sample in sample collection.”
Kll:05- “Training is needed also for a refresher course.”
Kll:05- “Educate the farmers on the importance of ethics.”
Kll:05- “We need sensitisation of farmers to avoid self-treatment.”

2. Funding and investment
Kll:05- “In Nakaseke, we don’t have private labs.”

3. By-laws and policies

N/A

Kll:06
Extracted quotations

Theme 1) Livestock - poultry diseases and clientele

Codes
1. Livestock - poultry diseases and animal losses
Kll:06- “CBPP, Brucellosis, PPR, African Swine Fever, Avian diseases.”

2. Clientele
Kll:06- “Farmers, followed by vets as clientele has relaxed since there has been shortage of labs.”

Theme 2) Challenges towards existing animal diagnostic services

Codes
1. Access to labs
Kll:06- “Availability and coverage of labs is still lacking. They cannot serve all clients, there at least should be a lab at each district.”
Kll:06- “Someone taking samples from upcountry is too long.”
Kll:06- “Where are they? I don’t know veterinary labs which are in place apart from the ones Zoetis has put in place like Ssembabule, Nakaseke. One lab by Dr. Musinguzi in Mbarara coming up.”
Kll:06- “Human labs are everywhere but vet labs are nowhere.”

2. Self-treatment on farms
Kll:06- “Vets treat by guessing trying several drugs, but consequences aren’t good.”

Kll:06- “Using diagnosis before treatment helps a vet to reduce cost of treatment and gain farmers trust.”
Kll:06- “Users have spent a long time without using labs, so gravitating to using their own perceptions in managing animal diseases and products is the new norm.”

3. Lab human resources
Kll:06- “We also dint have the right human resource even in numbers, in the district labs which don’t have personnel. The vet or DVO (district veterinary officer) is the lab technician. Even government labs there is lack of staff.”

Additional code

4. Laboratory equipment and supplies
Kll:06- “Our national labs level of equipment and staffing is lacking because at some point we constructed BSL3 lab (NADEC) but has never operated as one.”
Kll:06- “Lack of reagents sometimes there was a big outbreak of FMD including the presidents farm and we wanted to investigate but did not have the reagents. We panicked to buy from private sector, but the man said i have to order from UK and it took 3-4 months while there was quarantine on the presidents farm.”

<u>Theme 3) Education and Government policies</u>

Codes
1. Education and training
Kll:06- “Raise awareness amongst all vets and farmers and other potential lab users.”
Kll:06- “Collaboration with strategic partners for awareness and publicity, also collaboration with farmers and other vets go into the field together and engage in training, and practices.”
Kll:06- “Awareness campaigns, explain cost reduction, animal welfare issues, opportunities to access better markets, their own health impact on consuming animals on treatment.”
Kll:06- “Awareness and availing services closer to them to point closer other than MUK. Tell them professional impact and service delivering, subsidising for vets too.”

2. Funding and investment
Kll:06- “The funding support is definitely quite inadequate, and depending mainly on donors like FAO and well-wishers.”
Kll:06- “Services remain free, but as the country to commercialise and private sector has become commercialised, we should start charging for these services.”
Kll:06- “There was a time when government was giving free services when we convinced farmers to adapt to new technologies and getting improved breeds but now we have passed that phase and the demand for services has increased for government to continue to give free services.”
Kll:06- “We have only Chemiphar (a private ISO accredited lab for chemical residue analysis) which is private and very expensive and UNBS (Uganda National Bureau of Standards).”
Kll:06- “As the animal industries commercialise we need private labs, because demand is high as well as trade requirements where we have to manage a whole value chain.”
Kll:06- “We need a low, and we are going to draft animal diseases pact to compel use of labs for farmers and vets. We are beginning to draft using money under a project. I was putting my pieces together of what should be under the law and compelling use of labs.”

3. By-laws and policies
Kll:06- “Currently people in regulation need to have accredited labs such that services can be trusted by everybody, but we don’t have.”
Kll:06- “Accreditation is a big setback, accreditation is failing.”
Kll:06- “We need a law for our lab regulation and government cost recovery.”

Kll:07
Extracted quotations

Theme 1) Livestock - poultry diseases and clientele

Codes
1. Livestock - poultry diseases and animal losses
Kll:07- “Fertility and production diseases like brucellosis, those ones constitute a greater percentage our serology.”
Kll:07- “Transboundary animal diseases we do very well like FMD, Anthrax, Rift Valley Fever, CBPP, Swine diseases (ASF and all the other Hemorrhagic Syndromes, CSF, BSE and porcine erysipelas) except rabies.”

2. Clientele
Kll:07- “Farmers submitting samples through the districts, mainly commercial farmers maybe medium scale.”
Kll:07- “NGOs and CBOs like mercy corps in Karamoja. Partners like FAO and pharmaceuticals.”
Kll:07- “

Theme 2) Challenges towards existing animal diagnostic services

Codes
1. Access to labs
Kll:07- “If you look at the coverage and also demands like other districts there is minimum demand, if you consider other regions. There is more demand in the West and Central and then less of other regions, so I think we would be interested to extend and may be explore more what happens to the local person in other regions. They are not demanding for this or they are not aware of the services.”
Kll:07- “The challenge with the management of our labs, is that they are located within the districts and they are managed but local government, management is not so good.”
Kll:07- “District labs are everywhere in the district and over 30 labs in the traditional old districts, with new districts coming so how a lab is established depends on how the DVO lobbies.”
Kll:07- “It is also about extending diagnostic services at the local level because that is where our huge clients are, if it is a farmer level we should reach down because how many farmers can come here for services?”

2. Self-treatment on farms
Kll:07- “Some vet services do extension and treatment, I think we should encourage them to back up their work with the laboratory findings i think that would be good, but we still should also train them on how to get the right samples of the test. Because I know some of them have drugs, so they treat before diagnosis.”

3. Lab human resources
Kll:07- "Well equipped also in terms of staff, we participate in proficiency testing and we perform well. We always do a wide range of PTs."
Kll:07- "We have other regional labs in Fortportal, Arua, Gulu, Lira but have been constrained by lack of technicians, DVOs and the ones managing the labs."

Additional code

4. Laboratory equipment and supplies
Kll:07- "The National referral lab is really well equipped and even some of the supplies are still wrapped can't be used due to space."
Kll:07- "We supply them with kits for serology but still as I told you the services are free, so we do not meet their needs 100%."
Kll:07- "Most of our needs are met through the development partners as I have told you because they help in that. Development partners like FAO offer support such as reagents, test kits, sampling equipment, the cold chain and staff capacity building. The routine maintenance and calibration of equipment is within the Ministry."

Theme 3) Education and Government policies

Codes
1. Education and training
Kll:07- "What I know is that we are yet to do a lot in terms of veterinary diagnostics, in terms of creating awareness, demand and more training."
Kll:07- "First we should create more awareness for diagnostic services."
Kll:07- "We could do open days for labs in the country."
Kll:07- "We should provide awareness about the test range done in the lab similar to vaccination schedules given by poultry breeders to farmers such that they can know what to test for."
Kll:07- "Engage other local leaders like community leaders, churches etc., when we are creating awareness."
Kll:07- "I think academic and research institutions should teach on diagnostics, we know that there are a lot of samples coming into our Central diagnostic laboratory (CDL) here (COVAB), so that is those academic institutions. You should look at what those institutions do and how best we can harmonise and come up with the whole picture of the country or a particular region."

2. Funding and investment
Kll:07- "The services are still free and you know diagnostics is expensive so we need to still engage with them and start sharing the costs. Free service is not sustainable the farmer may need to start paying for the services."
Kll:07- "In my opinion, veterinary diagnostic laboratories are very expensive to establish, and in terms of payment for the service, if you are to attract more private people then it should be profitable to them, of which currently we are not able to have an effective demand to meet the interest of the private sector."
Kll:07- "I think the issue is demanding, firstly I think generally the public should demand for quality products. The quality products may be certified or proven or re branded you know,

those branding of products like this is High premium and grading. So if this ties in with market demands they will definitely build more laboratories and that is where the private laboratories will come in handy meeting the demand."
Kll:07- "I think some of the tests are quite expensive compared to the value of the animal, quite expensive. You weigh a goat of 20 Kilos, and spend 100000ugx to treat it, so first of all to test and then prescribe are done to guide the vet but it is not worth it given that the animal does not live for long."
Kll:07- "Clients should be willing to pay , there should be some prices tagged to the product, but also that depends on whether they are getting profits for their products and services like an input of one is the output of the other."
Kll:07- "I know there is greed for money, so there is need to remind them for professional ethics."

3. By-laws and policies
Kll:07- "We have control surveillance programs for mandatory report on diseases outbreaks, we have to notify the DVOs in Karamoja and refugee camps dealing with recovery programs."
KLL:07- "Within East African community we have cross-border collaborations especially for management of Transboundary animal diseases, so they have been key in that."
Kll:07- "When it is an outbreak, those are within the legal framework, it is mandatory, they are supposed to test per farm and then issue control measures, animal movement control or put in quarantine."
Kll:07- "Currently we do not have the vet lab diagnostic policy that governs the country. This leaves a lot of dilemma, we need standards so all service providers in terms of diagnostics should conform to a certain standard. We should do annual monitoring, and check they are all registered."
Kll:07- "We should also sensitise the community to demand quality products because that is where we go backward, we should have standards. The veterinary laboratory policy should sensitise and organise vet diagnostic services."
Kll:07- "We do not have accreditation that is what we should really strive for."

Kll:08
Extracted quotations

Theme 1) Livestock - poultry diseases and clientele

Codes
1. Livestock - poultry diseases and animal losses
Kll:08- "Infectious diseases such as ECF, Trypanosomosis, Leptospirosis, Mastitis, fertility related diseases.

2. Clientele
Kll:08- "Farmers, and mainly commercial farmers."

Theme 2) Challenges towards existing animal diagnostic services

Codes
1. Access to labs

Kll:08- “There are very few veterinary diagnostic services, a few of them exist but not well distributed, they are skewed in their distribution.”
Kll:08- “The distribution is poor and quite inadequate and of course in many places it is not available.”
Kll:08- “The users are few, even the professionals who would want to use them are scattered and are not within reach in these locations to use them.

2. Self-treatment on farms

N/A

3. Lab human resources
Kll:08- “There is a big issue with human resource staffing and administrative support, you can see how the lab in Mbarara is struggling and how they had to evict the services from the main town to outside.”
Kll:08- “If we had really good professionals and these non-para vets who are there, and they are really following good laws, farmers would be encouraged.”

Additional code

4. Laboratory equipment and supplies
Kll:08- “The equipment is ancient equipment, not modern and the scanty competent human resource.”
Kll:08- “Yes, some standards are ok, but I think there are some places where there is insufficient equipment. There is something missing which is beyond the laboratory capacities that exist here.”

<u>Theme 3) Education and Government policies</u>

Codes
1. Education and training - government/ accreditation
Kll:08- “Train people, CPD, and hands on.”

2. Funding and investment
Kll:08- “The prices of the commodities produced on livestock farms are quite discouraging so if they are not making good money from it farmers cannot pay good price.”
Kll:08- “The money out of this service delivery in the veterinary sector is not encouraging.”
Kll:08- “The training of the personnel is not encouraging; the employment opportunities do not encourage people to invest in training to support these services in the lab field.”
Kll:08- ‘Privatised veterinary services do not exist in this country. So, if you have a private service, you can even establish a small lab there, and there are no labs there because people cannot pay, so you can’t get enough money to attract clients. For example, when professor (NAME) faculty member at COVAB came back from his masters, he started his private veterinary service in Makindye, but it collapsed, and he had to come back to Makerere.”

3. By-laws and policies
Kll:08- “I do not think farmers would refuse to pay as long as it is the requirement.”
Kll:08- “Private sector to invest in this because it does not pay in short or medium terms. That is a long-term thing, investing in a long term here is a bit difficult.”

Kll:08- “The political environment does not appreciate the input; they do not know the results from laboratory diagnostics.”
Kll:08- “If they know that they cannot access them unless by following the regulations (prescription), they have by the professional law.”
Kll:08- “Before giving the drug to the farmer, the drug needs to be endorsed by a person who is on the register (licensed veterinarian).”
Kll:08- “Policy regulations on vet lab and general veterinary service delivery and also have referral system.”
Kll:08- “government policy that would require results from diagnostic services for certification of some products from livestock and food that is on the market.”
